# Hormonal axes in *Drosophila:* regulation of hormone release and multiplicity of actions

**DOI:** 10.1007/s00441-020-03264-z

**Published:** 2020-08-22

**Authors:** Dick R. Nässel, Meet Zandawala

**Affiliations:** 1 Department of Zoology, Stockholm University, Stockholm, Sweden; 2 Department of Neuroscience, Brown University, Providence, RI USA

**Keywords:** Insect brain, Insect neurosecretory cells, Peptide hormones, Neuropeptides, Insulin signaling

## Abstract

Hormones regulate development, as well as many vital processes in the daily life of an animal. Many of these hormones are peptides that act at a higher hierarchical level in the animal with roles as organizers that globally orchestrate metabolism, physiology and behavior. Peptide hormones can act on multiple peripheral targets and simultaneously convey basal states, such as metabolic status and sleep-awake or arousal across many central neuronal circuits. Thereby, they coordinate responses to changing internal and external environments. The activity of neurosecretory cells is controlled either by (1) cell autonomous sensors, or (2) by other neurons that relay signals from sensors in peripheral tissues and (3) by feedback from target cells. Thus, a hormonal signaling axis commonly comprises several components. In mammals and other vertebrates, several hormonal axes are known, such as the hypothalamic-pituitary-gonad axis or the hypothalamic-pituitary-thyroid axis that regulate reproduction and metabolism, respectively. It has been proposed that the basic organization of such hormonal axes is evolutionarily old and that cellular homologs of the hypothalamic-pituitary system can be found for instance in insects. To obtain an appreciation of the similarities between insect and vertebrate neurosecretory axes, we review the organization of neurosecretory cell systems in *Drosophila*. Our review outlines the major peptidergic hormonal pathways known in *Drosophila* and presents a set of schemes of hormonal axes and orchestrating peptidergic systems. The detailed organization of the larval and adult *Drosophila* neurosecretory systems displays only very basic similarities to those in other arthropods and vertebrates.

**Electronic supplementary material:**

The online version of this article (10.1007/s00441-020-03264-z) contains supplementary material, which is available to authorized users.

## Introduction

Hormonal signaling regulates developmental processes as well as most aspects of physiology and behavior in the daily life of animals (Elphick et al. 2018; Jékely et al. 2018; Nässel and Zandawala 2019; Norris 1997; Schoofs et al. 2017; Strand 1999; Takei et al. 2015). Many of these hormones are peptides of a great variety. Both in protostomes (invertebrates) and in deuterostomes (invertebrates and vertebrates), the number of different peptide-encoding genes is high (Elphick et al. 2018; Jekely 2013; Mirabeau and Joly 2013), ranging from about 50 in the fly *Drosophila melanogaster* (Hewes and Taghert 2001; Nässel and Zandawala 2019; Vanden Broeck 2001) to more than 100 in the nematode worm *Caenorhabditis elegans* (Husson et al. 2007; Li and Kim 2008) and humans (See Database^1^). However, only a small portion of these is known to act as bona fide circulating hormones. Yet, peptide hormone signaling is diverse and complex and differs over the life cycle.

Peptide hormones commonly act at a high hierarchical level in the animal and play roles as organizers that globally orchestrate various aspects of development, physiology and behavior (Hauser et al. 2006; Jékely et al. 2018; Kim et al. 2017; Nagata and Zhou 2019; Nässel et al. 2019; Nässel and Zandawala 2019; Owusu-Ansah and Perrimon 2015; Schoofs et al. 2017; Taghert and Nitabach 2012). In adult physiology, hormones can act on several peripheral targets and simultaneously convey basal organismal states, such as metabolic status, sleep-awake, or arousal, across many central neuronal circuits and thus orchestrate responses to changing internal or external environments (see Jékely et al. 2018; Kim et al. 2017; Martelli et al. 2017; Nässel et al. 2019; Nässel and Zandawala 2019; Schoofs et al. 2017).

The activity of neurosecretory cells that release peptide hormones is controlled by other neurons that relay signals from peripheral sensors or by feedback from peripheral target cells (Ahmad et al. 2019; Delgado et al. 2017; Nässel et al. 2013; Nässel and Vanden Broeck 2016; Romanov et al. 2019; Zavala et al. 2019). Some neurosecretory and endocrine cells possess autonomous sensors that monitor nutritional or energy states to control hormone release (Kim and Rulifson 2004; Nässel and Zandawala 2019; Park et al. 2016; Park et al. 2014). Other controls of neurosecretory cells are constituted by central circuits that orchestrate basal states, or by inputs from the biological clock (Ahmad et al. 2019; Nagy et al. 2019a; Nässel et al. 2019; Nässel and Zandawala 2019; Selcho et al. 2017). After hormone release, target cells can respond by releasing feedback signals to the neurosecretory cells. Thus, a hormonal signaling axis commonly comprises several components and is not unidirectional. In mammals and other vertebrates, several hormonal axes are known, for example, the hypothalamic-pituitary-gonad (HPG) axis or the hypothalamic-pituitary-thyroid (HPT) axis that regulate reproduction and metabolism, respectively (see Le Tissier et al. 2017; Nässel and Larhammar 2013; Norris 1997; Zavala et al. 2019). Each of these is complex with different levels of regulation and feedback.

It has been proposed that the organization of hormonal axes is evolutionarily old (Hartenstein 2006; Raabe 1989; Scharrer 1987) and that cellular homologs of the hypothalamic-pituitary system can be found for instance in the vinegar fly *Drosophila melanogaster* (de Velasco et al. 2007; De Velasco et al. 2004; Park et al. 2011; Wang et al. 2007) and the polychaete worm *Platynereis dumerilii* (Tessmar-Raible 2007; Tessmar-Raible et al. 2007). These proposals were originally based on anatomical similarities in organization and more recently on the expression of a number of transcription factors and hormones in secretory cells that are shared by mammals and these invertebrates but also on the general organization of the neurosecretory cells and their release sites, at least during embryonic development. Thus, the embryonic origin of some neurosecretory cells seems to be conserved over evolution, yet when looking more carefully at the detailed organization of the vertebrate hormonal axes and those in insects and worms in postembryonic animals, the similarities are not so obvious. For instance, in insects, there are two major groups of brain neurosecretory cells, the lateral neurosecretory cells (LNCs) and the median neurosecretory cells (MNCs), each of which comprises a relatively small number of cells (see Hartenstein 2006; Raabe 1989), generating a rather limited number of peptide hormones (see Nässel and Zandawala 2019). The areas where LNCs and MNCs are located are designated pars lateralis (PL) and pars intercerebralis (PI), respectively. Both LNCs and MNCs have axons that terminate in peripheral release sites in contact with the circulation or with secretory cells that produce additional hormones. Two such termination sites are the corpora cardiaca (CC) and corpora allata (CA) that reside in contact with the anterior blood vessel, the “aorta.” In some insects, like *Drosophila,* the CC and CA are very small and axon terminations from LNCs and MNCs spread out along the anterior aorta as well as the foregut and crop duct (Nässel and Zandawala 2019; Rulifson et al. 2002). In the PL and PI area, there are also other neurons, some of which secrete release-regulating factors that act on the neurosecretory cells, while others seem to be interneurons. One example of PI interneurons with widespread arborizations and actions on multiple target circuits is four neurons producing the neuropeptide SIFamide (SIFa) (Martelli et al. 2017; Terhzaz et al. 2007). We will deal with the roles of these SIFa neurons and other interneurons as well as bona fide neurosecretory cells, in orchestrating physiology and behavior.

To get a better appreciation of the similarities between insect and vertebrate neurosecretory axes, we review here the organization of neurosecretory cells in *Drosophila* and how they are integrated in “signaling circuits” (or axes) from sensory inputs, releasing factors, hormonal targets and feedback. These hormonal regulatory circuits described are commonly based on both anatomical and functional analyses but in some cases only anatomical data are available. In assembling this summary, we also came across several peptidergic systems that are not releasing neurohormones into the circulation but still playing roles in integrating behavior and/or physiology in a global fashion. One example is the SIFa neurons mentioned above. Thus, we summarize both circuits using bona fide peptide hormones and those where peptides act globally by paracrine (and/or synaptic) signaling within the central nervous system (CNS). We also present data on neurosecretory cell systems in the ventral nerve cord and enteroendocrine cells of the intestine.

Our review outlines the major peptidergic hormonal pathways known in *Drosophila* (and other insects) and presents a set of schemes of hormonal axes. We find that the *Drosophila* neurosecretory systems, although embryologically somewhat similar to those of vertebrates or annelid worms, are quite divergent in larvae and adults and display an organization that deviates from the pattern seen in the other phyla.

## Definitions of terms used in this review

**Fig. 1:**
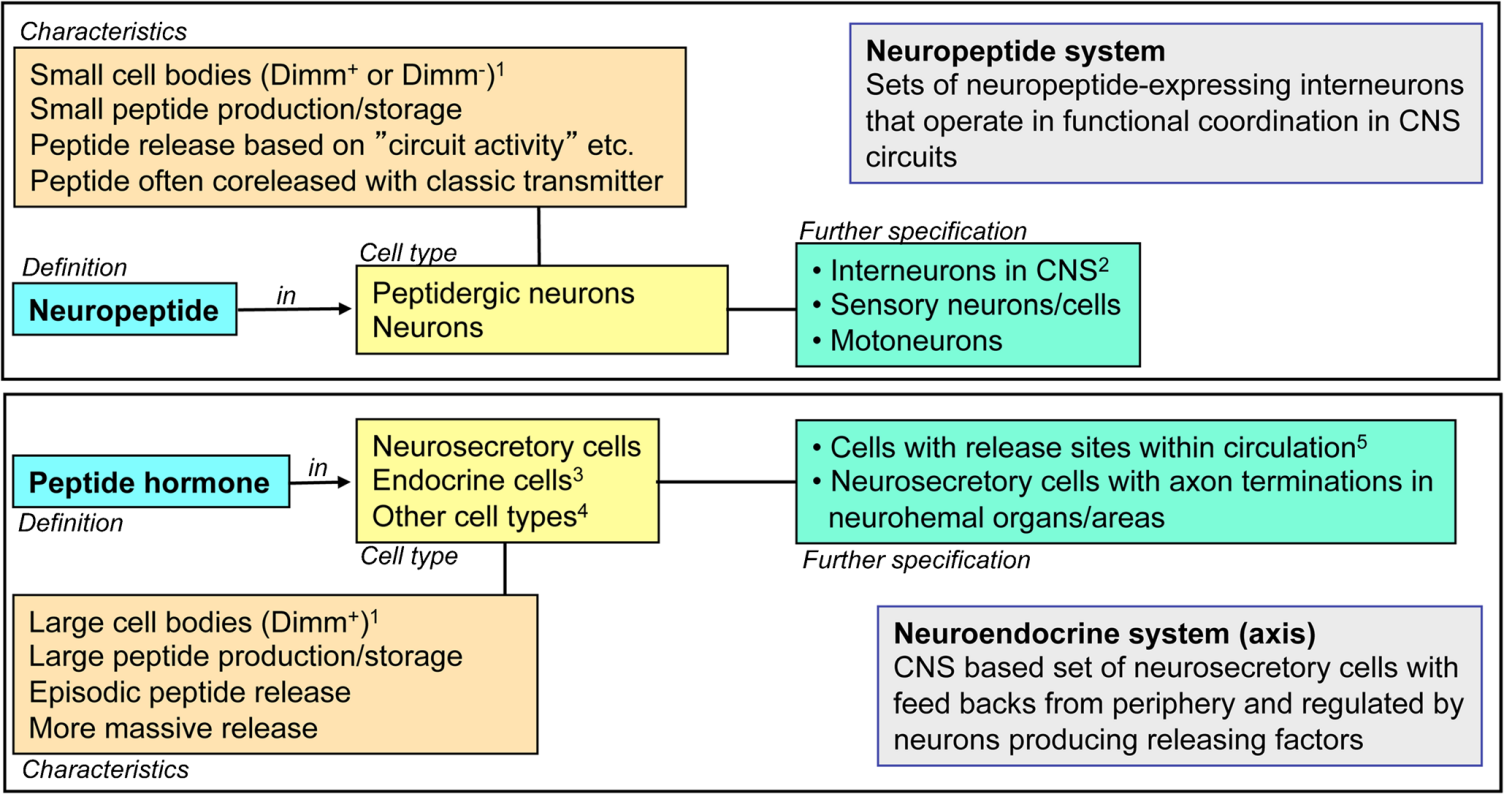
Definitions of terms used in this review. The top box shows definitions and characteristics of neuropeptide and peptidergic neurons. Dimm (Dimmed) is a transcription factor that is known to specify many, especially larger, peptidergic neurons and neuroendocrine cells (Hewes et al. 2003). The lower box defines peptide hormone and neurosecretory (endocrine) cells. A given peptide can be both a neuropeptide and peptide hormone; thus, a specific GPCR can be activated by both a neuropeptide (at close range) and a peptide hormone (via circulation), probably at different EC_50_ values. Notes: ^1^Dimm expression is predominantly seen in larger neurons. ^2^Including neurons producing hormone release-regulating factors. ^3^And/or secretory cells. ^4^Muscle cells, gland cells and epithelial cells. ^5^Or other body cavities in certain invertebrates

Here we first outline the terms/definitions we are using (Fig. 1). We distinguish here between neuropeptide and peptide hormone. Neuropeptides act at a closer range after release from peptidergic neurons (inter-, sensory-, or motor neurons) and peptide hormones are released into the circulation from neurosecretory cells or endocrine cells. Thus, there are bona fide neuroendocrine (neurosecretory) systems acting systemically (peptide hormones) via the circulation and interneuronal neuropeptide systems acting within the CNS by synaptic (by means of small molecule co-transmitters) or paracrine peptidergic signaling (see Nässel 2009; Zupanc 1996). Some of the peptidergic systems encompass a small number of globally arborizing neurons, such as the four SIFamide (SIFa)-producing brain neurons in *Drosophila* (Martelli et al. 2017; Terhzaz et al. 2007; Verleyen et al. 2004). The neurosecretory cells may additionally signal synaptically with other neurons within the CNS, possibly with small molecule neurotransmitters (see Nässel 2018; Schlegel et al. 2016). Neurons/cells of both types of system can utilize more than one neuropeptide/peptide hormone or even colocalized small molecule neurotransmitters (Hökfelt et al. 2018; Hökfelt et al. 1987; Nässel 2018; Nusbaum et al. 2017; Svensson et al. 2019). Furthermore, it has been shown in *Drosophila* that peptide hormones such as insulin-like peptides (ILPs) released from brain neurosecretory cells (insulin-producing cells; IPCs) act not only as circulating hormones on peripheral targets but also signal to brain neurons, probably in a paracrine fashion (Bader et al. 2013).

The activity of neurosecretory cells is controlled by various neurons, or by secreted factors from peripheral cells and tissues that relay signals mediating information about the internal and external environment (Ahmad et al. 2019; Lin et al. 2019; Nässel and Vanden Broeck 2016; Nässel and Zandawala 2019; Owusu-Ansah and Perrimon 2014, 2015; Rajan and Perrimon 2011). We will discuss these neuronal pathways and the release-inducing and inhibiting factors they utilize, as well as interorgan signaling.

Finally, some of the neurosecretory cells (or even peptidergic neurons) have been shown to be intrinsically nutrient sensing (Dus et al. 2015; Kreneisz et al. 2010; Oh et al. 2019; Park et al. 2014; Yang et al. 2018; Yurgel et al. 2019); others receive signals from nutrient-sensing cells in the intestine or fat body (Alfa et al. 2015; Geminard et al. 2009; Kwak et al. 2013; Nässel and Zandawala 2019; Rajan and Perrimon 2012; Ren et al. 2015; Sun et al. 2017) and this mechanism is utilized for regulation of hormone release. The peptides discussed in this review are listed in Table 1, which also provides a list of the acronyms used in the text and figures.

**Table 1:** Peptide acronyms, precursor genes and peptide functions in *Drosophila*. Excluding gut EECs (instead, see Table 2). Note that this is not a comprehensive list of *Drosophila* peptides but a list of peptides discussed in this review

Acronym	Peptide name	Precursor gene^1^	Function^2^
AKH	Adipokinetic hormone	CG1171	Hormone, NRF
AstA	Allatostatin A	CG13633	Hormone, NRF
AstB/MIP	Allatostatin B	CG6456	NM
AstC	Allatostatin C	CG14919	NM^3^
Burs α	Bursicon α	CG13419	Hormone, NRF
CAPA	Capability (capa 1, 2, PK 1)	CG15520	Hormone
CCAP	Crustacean cardioactive peptide	CG4910	NM
CCHa1	CCHamide 1	CG14358	Hormone^3^, NM
CCHa2	CCHamide 2	CG14375	Hormone, NRF
CRZ	Corazonin	CG3302	Hormone, NRF
DH31	Diuretic hormone 31	CG13094	Hormone, NM
DH44	Diuretic hormone 44	CG8348	Hormone, NM
DMS	*Drosophila* myosuppressin	CG6440	NM^4^
DILP1–8	*Drosophila* insulin-like peptides	See note^5^	Hormone, NRF^6^
DSK	*Drosophila* sulfakinin	CG18090	Hormone^7^, NM
EH	Eclosion hormone	CG5400	Hormone, NM
ETH	Ecdysis-triggering hormone	CG18105	Hormone, NRF
Hugin-PK	Hugin-pyrokinin (PK 2)	CG6371	Hormone, NRF
ITP	Ion transport peptide	CG13586	Hormone, NM
LK	Leucokinin	CG13480	Hormone, NRF
Lst	Limostatin	CG8317	NRF
NPF	Neuropeptide F	CG10342	NM, NRF
OK-B	Orcokinin B	CG13565	Hormone^3^, NM
PDF	Pigment-dispersing factor	CG6496	Hormone, NRF
PTTH	Prothoracicotropic hormone	CG13687	Hormone, NRF
RYa	RYamide	CG40733	Hormone
SIFa	SIFamide	CG4681	NM
sNPF	Short neuropeptide F	CG13968	NRF
TK	Tachykinin	CG14734	NRF

^1^Gene annotation

^2^Specifies whether hormone, neuromodulator (NM), or neuromodulator and release-regulating factor (NRF). Note that hormonal functions are in several cases inferred from indirect evidence. For details, see Nässel and Zandawala et al. (2019)

^3^Not clear whether also hormone

^4^Acts on crop and may also be released as a hormone (hormonal function not yet established)

^5^DILP1 = CG13173, DILP2 = CG8167, DILP3 = CG14167, DILP4 = CG6736, DILP5 = CG33273, DILP6 = CG14049, DILP7 = CG13317, DILP8 = CG14059

^6^The DILPs have different functions; all are hormones but only some (e.g., DILP2 and DILP7) appear to act as neuromodulators or release-regulating factors

^7^Although DSK has been identified in IPCs, its presence in neurohemal areas associated with the brain has not been determined. Yet a possible hormonal role has been proposed (Söderberg et al. 2012)

## Organization of the neuroendocrine system in the fly brain compared to that of mammals

**Fig. 2:**
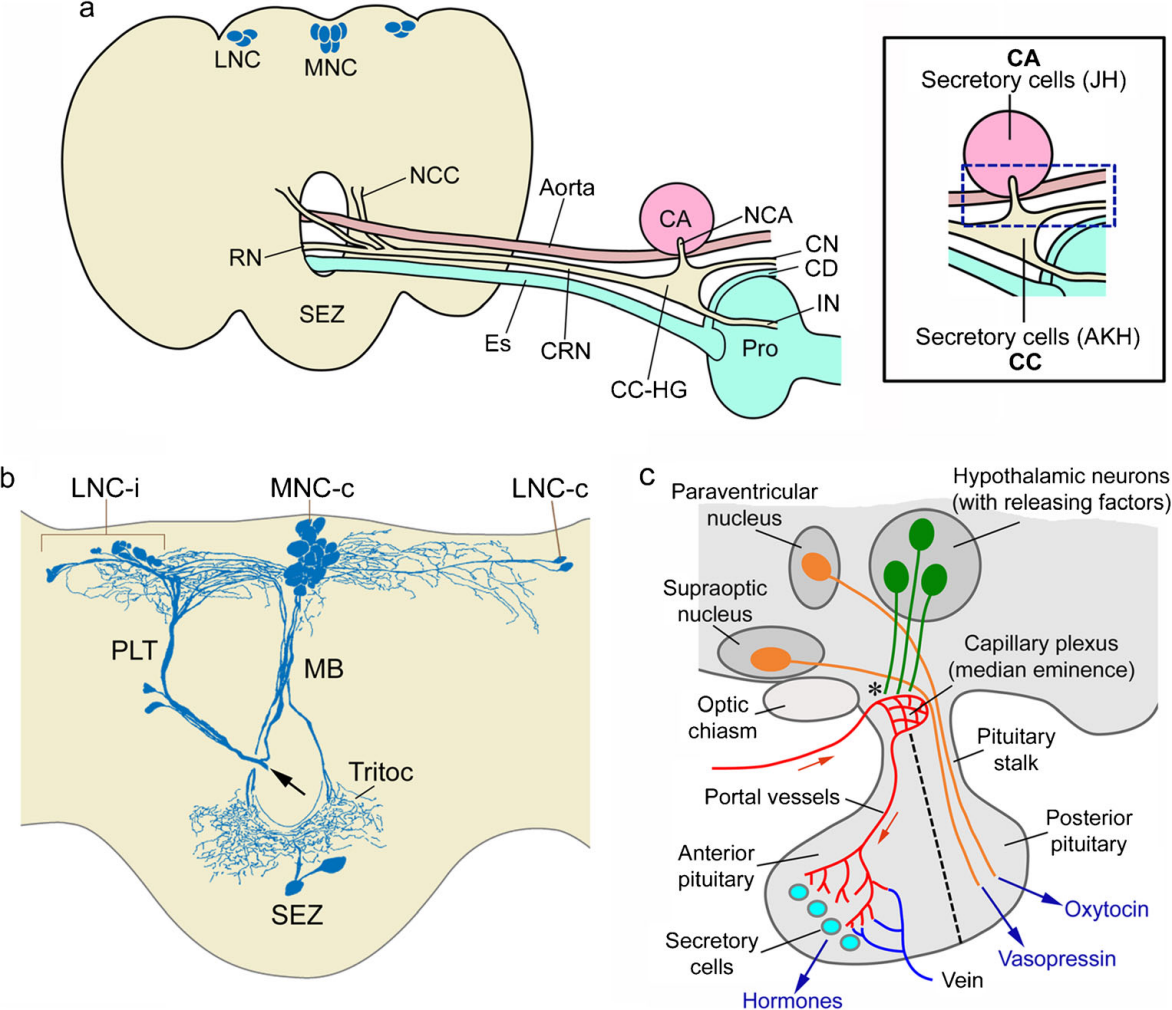
Organization of the neuroendocrine system in the fly brain compared to mammals*.* **a** Schematic of the brain and retrocerebral complex in flies. Lateral (LNC) and median (MNC) neurosecretory cell groups send their axons via a pair of nerves (nervii corpora cardiaca, NCC) to the fused CRN (cardiaca-recurrent nerve), which supplies the corpora cardiaca (CC) and hypocerebral ganglion (HG), as well as the corpora allata (CA) via the paired NCA (nervii corpora allata) nerves. Axons continue via the HG into the crop nerve (CN), which follows the crop duct (CD) and branch over the crop and an intestinal nerve (IN) that supplies branches over the proventriculus (Pro) and anterior midgut. These nerve branches contain axon terminations of the LNCs, MNCs and subesophageal zone (SEZ) neurosecretory cells and form neurohemal release sites/areas. Axon terminations from these cells are also found along the aorta. Other abbreviations: Es, esophagus; RN, recurrent nerve. The inset shows an enlarged view of the CC-CA region, indicating that both contain secretory cells producing hormones, adipokinetic hormone (AKH) and juvenile hormone (JH). The blue box indicates the area where axon terminations of LNCs and MNCs are in contact with the circulation (neurohemal area). **b** Frontal view of a blowfly brain where one NCC was backfilled with cobalt chloride (at the arrow) into the posterior lateral tract (PLT) and median bundle (MB). Neurosecretory cells were revealed in contralateral MNCs (MNC-c) and LNCs (LNC-c) as well as ipsilateral LNCs (LNC-i) and the SEZ. Note extensive overlapping arborizations dorsally and in tritocerebrum, Tritoc. These two figures (**a**, **b**) were redrawn and slightly altered from Shiga et al. (2000) with permission from Dr. Sakiko Shiga. Panel **b** appeared in a similar form in Nässel and Larhammar (2013). **c** Schematic of a generalized mammalian hypothalamus-pituitary. The asterisk indicates where hormone release–regulating factors enter the capillary plexus of the portal system (“neurohemal” area). Hormone-producing secretory cells are found in the anterior pituitary and the hormones released into the circulation (see text for hormones). A more detailed scheme is shown in Fig. 12

The insect neuroendocrine system was first explored by neurosecretion staining techniques and it was pointed out in these earlier studies that the organization of LNC, MNC, CC and CA in insects displays general similarities to the vertebrate hypothalamus and pituitary (see (Raabe 1989; Scharrer 1987; Scharrer and Scharrer 1963). In very general terms, this may still be valid to an extent in that the embryonic origin/lineage of neurosecretory cells and the expression of certain transcription factors in these cells during development show some similarities (Clements et al. 2008; de Velasco et al. 2007; Hartenstein 2006; Wang et al. 2007; see also Tessmar-Raible 2007; Tessmar-Raible et al. 2007). However, when analyzing the detailed anatomical organization in adults, as well as the complement of peptide hormones and other signaling compounds, the similarities are somewhat less obvious. Thus, during postembryonic development, the neurosecretory systems differentiate to different degrees to display taxon-specific differences in complexities. Furthermore, the number of peptides in the neurosecretory systems that are conserved during evolution is relatively small. We shall get back to details of the similarities and differences towards the end of the review but show the general organization of fly and mammalian neurosecretory systems in Fig. 2 to remind the reader. Note that in contrast to mammals, insects do not have a closed circulatory system. Instead they possess an open-ended dorsal vessel (consisting of an anterior aorta and a posterior contractile heart) aiding in circulating the hemolymph (the insect equivalent of blood) through the body cavities, or coelom (see Hillyer and Pass 2020).

The fly brain and associated neurosecretory cells, endocrine organs and neurohemal release sites are shown in Fig. 2(a, b). The cephalic neurosecretory cells in insects, including *Drosophila*, are arranged in three major parts of the brain, the MNC, LNC and subesophageal zone (SEZ), also known as gnathal ganglia. Secretory cells in the CC produce the peptides adipokinetic hormone (AKH) and limostatin (Lst) and cells in the CA produce juvenile hormone (JH), a sesquiterpenoid (Fig. 2a, inset). Release of hormones occurs from axon terminations and endocrine cells in specific regions, neurohemal areas, in contact with the open circulation (Fig. 2a inset). The organization of the mammalian hypothalamus-pituitary is shown for comparison in Fig. 2(c). Note that in mammals, the factors regulating release act via the circulation (a capillary plexus) on the secretory cells in the anterior pituitary, whereas in insects such capillaries do not exist and regulation is by neurons/neuroendocrine cells contacting secretory cells in the brain or CC/CA. We discuss the release-regulating factors and hormones, as well as neurosecretory cell systems in the ventral nerve cord and periphery, including intestinal endocrine cells in later sections.

## Distribution of peptide hormones in neurosecretory cells in the adult *Drosophila* brain

**Fig. 3:**
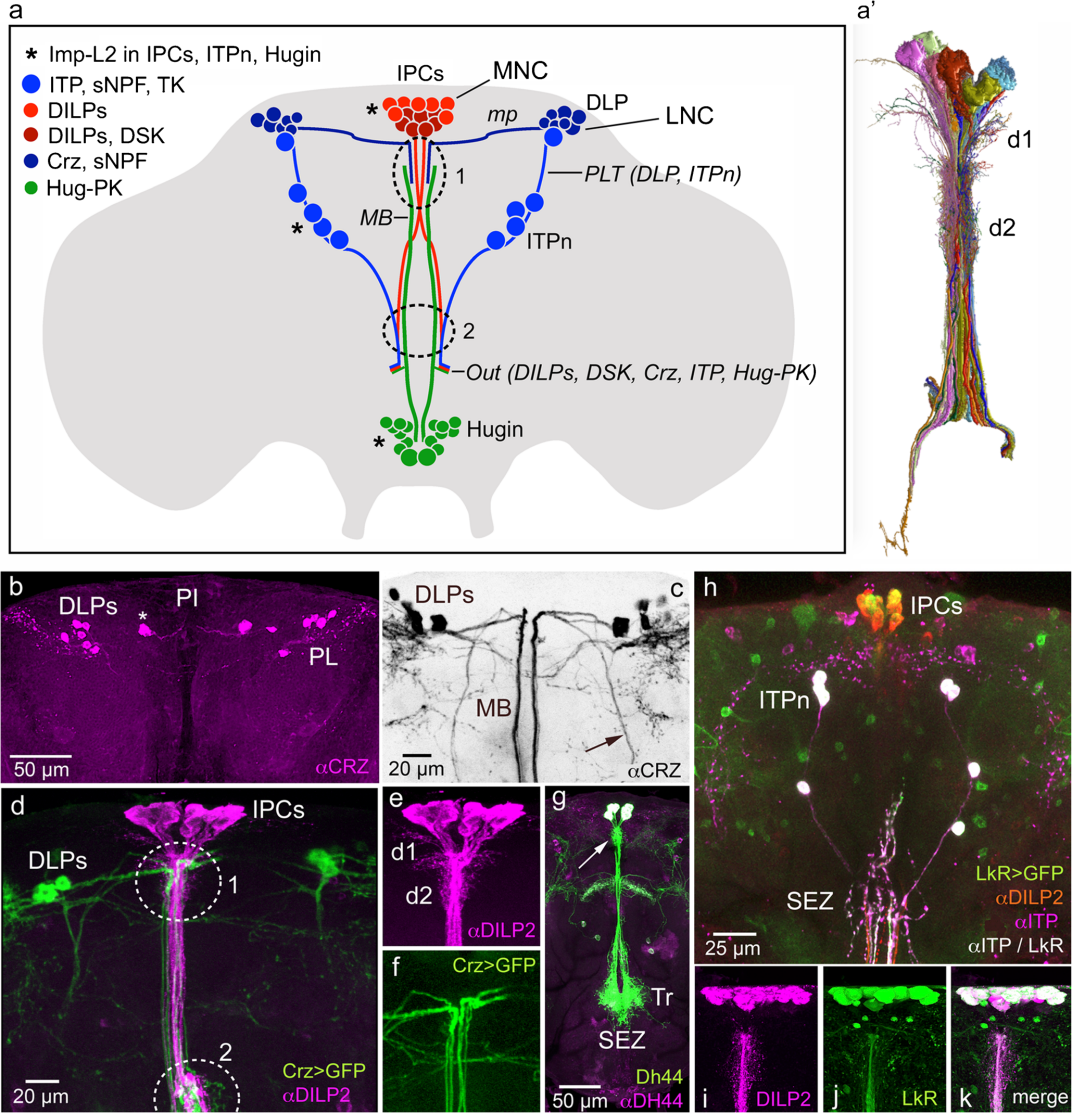
Distribution of a selection of peptide hormones in neurosecretory cells in the adult Drosophila brain. (a) Schematic of some of the lateral (LNC) and median (MNC) neurosecretory cell systems in the adult fly brain. Two sets of LNCs are shown: the DLPs that produce corazonin (CRZ) and short neuropeptide F (sNPF) (Kapan et al. 2012) and the ITPn that produce ion transport peptide (ITP), sNPF and tachykinin (TK) (Kahsai et al. 2010). One set of MNCs is depicted, the insulin-producing cells (IPCs) that produce insulin-like peptides (DILP1, 2, 3 and 5) of which a subpopulation also produces sulfakinin (DSK) (Söderberg et al. 2012). A set of neuroendocrine cells (Hugin cells) in the subesophageal zone (SEZ) produces the peptide pyrokinin (Hug-PK); a subset of these constitutes neurosecretory cells (Melcher and Pankratz 2005). Axons from these four cell groups run via the median bundle (MB) or posterior lateral tract (PLT), exit the brain (at Out) via the NCC (nervii corpora cardiaca; not shown) and they reach the retrocerebral complex where axon terminations are in contact with the circulation. The DLPs utilize sNPF as a factor that regulates release of DILPs and AKH (Kapan et al. 2012; Oh et al. 2019) and the ITPn possibly use TK and sNPF as releasing factors and Hugin cells may employ Hug-PK to regulate release of other peptides (Schlegel et al. 2016). Interactions between the cell systems shown probably occur at the sites indicated by 1 and 2. It was shown that three of these cell types (marked with asterisks) express the protein ImpL2 and that the IPCs signal to the ITPn and Hugin cells with DILP2 via the insulin receptor in an Imp-L2-dependent fashion (Bader et al. 2013). mp, medially projecting axons of LNCs. (a’) A set of MNCs (including IPCs) reconstructed from serial electron microscopic sections of *Drosophila* hemibrain. d1 and d2 two sets of presumed dendrites. This figure was compiled from data in neuPRINT (https://neuprint.janelia.org) (Clements et al. 2020; Xu et al. 2020; Zheng et al. 2018). (b) There are seven pairs of CRZ expressing DLPs in pars lateralis (PL). Note that in this specimen one larger pair of cells (asterisk) is slightly dislocated medially towards pars intercerebralis (PI). (c) The DLPs (inverted image; CRZ immunolabeling, αCRZ) have axons running through the median bundle (MB) and a lateral tract (arrow). (d) The DLPs (green) impinge on the IPCs (magenta) in encircled areas 1 and 2. (e, f) Details of IPCs and branches from DLPs. D1 and d2, two sets of IPC dendrites. (g) Six median neurosecretory cells producing diuretic hormone 44 (DH44) have dendrites at the arrow and axon branches in the tritocerebrum (Tr) and subesophageal zone (SEZ) rendered by *Dh44*-Gal4-driven GFP and antiserum to DH44. (h) Triple labeling with antisera to ITP (magenta/white), DILP2 (red) and *LkR*-Gal4-driven GFP (leucokinin receptor; green) reveals ITP neurons (ITPn) and IPCs. This indicates that the LkR is expressed on both ITPn and IPCs. (i–k) The IPCs express the leucokinin receptor (LkR) seen with LkR-Gal4-driven GFP and anti-DILP2. Panel (a) is updated from Nässel et al. (2013) with data from several of the papers cited above. Panel (b) is from Kubrak et al. (2016), (c–f) are from Kapan et al. (2012), (g) from Zandawala et al. (2018a) and (h–k) from Zandawala et al. (2018b), with permission from the publishers

Peptides that have been identified in different neurosecretory cell groups of *Drosophila* are listed in Table 2 and some of these neurosecretory cells are shown in Fig. 3. Note that we list peptides such as short neuropeptide F (sNPF) and tachykinin (TK) without considering the fact that they are encoded on precursors that generate several paracopies (peptide isoforms). The total number of MNC/LNCs in the brain of *Drosophila* is not clear but is likely to be around 60 cells. However, the number of neurosecretory cells in *Drosophila* with identified peptide hormones is even smaller. In adult flies, the MNC group (both hemispheres) has 24 cells, of 3–4 types and the LNC group in one hemisphere has 12 cells (of 2–3 types). These cell types are based on content of peptides determined using immunohistochemical approaches, sometimes in combination with Gal4 expression. Recent advances in single-cell transcriptomics may alter these numbers of cell types in the near future, as we begin to identify subtypes within supposedly homogenous groups of cells (Davie et al. 2018; Trapnell 2015); see also next section. There are LNCs with (1) colocalized ion transport peptide (ITP), sNPF and TK (Kahsai et al. 2010) and (2) those with corazonin (CRZ) and sNPF (Diesner et al. 2018; Kapan et al. 2012) of which one subset also expresses a fructose receptor and another a glucose transporter (Miyamoto et al. 2012; Oh et al. 2019). MNCs of different types produce (1) diuretic hormone 44 (DH44) (Cabrero et al. 2002; Dus et al. 2015), (2) dromyosuppressin (DMS) (Nichols 2003) and (3) colocalized insulin-like peptides (DILP1, 2, 3, 5) (Brogiolo et al. 2001; Liu et al. 2016; Nässel and Vanden Broeck 2016) and (4) a subpopulation of these produce colocalized drosulfakinin (DSK) (Söderberg et al. 2012). As mentioned above, there are likely additional cells in each group where the peptide is yet to be identified since backfilling the cut axons of the nerve to CC-CA with tracer (e.g., cobalt chloride) renders more cells, at least in a blowfly species (Fig. 2b; Shiga et al. 2000). One set of subesophageal neurosecretory cells produce Hugin-PK (pyrokinin-2; PK-2) (Diesner et al. 2018; Melcher and Pankratz 2005; Neupert et al. 2007) and another set CAPA-PK (pyrokinin-1; PK-1) (Diesner et al. 2018; Kean et al. 2002). Details of several of these peptidergic neurosecretory cells are shown in Fig. 3(b–h). Some of the cells shown in Fig. 3(a) communicate with each other. Thus, it has been shown that the DLP neurons act on IPCs via sNPF to regulate DILP release (Kapan et al. 2012; Oh et al. 2019). The same cells release CRZ as a circulating hormone (Kubrak et al. 2016). This suggests that a set of cells can both control DILP release and liberate hormonal CRZ. In Fig. 3(b–f), the anatomical substrate for DLP action on IPCs is shown. Six MNCs that produce DH44 are shown in Fig. 3(g). The IPCs are also under control by neurons releasing leucokinin (LK) and both IPCs and ITP-producing neurons (ITPn) express LK receptor (Fig. 3h–k) (Zandawala et al. 2018b). Action of LK on IPCs has been demonstrated (Yurgel et al. 2019; Zandawala et al. 2018b) but so far we have no evidence for action on the ITPn. These were examples of some *Drosophila* hormones and factors regulating their release. We will get back to this in more detail below but we want to point out here that the distribution of neurons regulating MNCs and LNCs is spread out in the brain and not organized in clear modules as in mammals within the hypothalamus-pituitary axis (HP; see Fig. 2c). In the next section, we use the IPCs as an example of organization of a hormonal axis including release-regulating neurons and endocrine cells, as well as peripheral feedbacks and interorgan communication.

**Table 2:** Neuropeptides and peptide hormones in neurosecretory cells, endocrine cells and orchestrating neurons of *Drosophila*

Peptide	Cell/neuron	Region	Stage^1^	Function
Brain neurosecretory cells and peripheral secretory cells				
DILP1,2,3,5, DSK	IPCs	MNC/PI	L/A^2^	Metabolism, feeding, stress responses, reproduction
DH44, DILP2	DH44-PI^3^	MNC/PI	L/A	Feeding, osmotic homeostasis
DMS	DMSn	MNC/PI	L/A	Crop motility
CRZ, sNPF	DLP	LNC/PL	L/A	IPC and CC regulation, metabolism, stress responses
ITP, TK, sNPF	ITPn	LNC/PL	L/A^4^	Osmotic homeostasis, metabolism, stress responses
PTTH	PTTHn	LNC/PL	L/P^5^	Regulation of ecdysone production, light avoidance
Hugin-PK	Hugin cells	SEZ	L/A	Feeding, locomotion
CAPA	SEn	SEZ	L/A	?
EH	VM	Brain	L/P^5^	Ecdysis behavior, light avoidance
AKH	CC	CC	L/A	Metabolism, food seeking
Lst	CC	CC	A	Metabolism, food seeking
DILP6	Adipocyte	Fat body	L/A	Growth, IPC regulation
CCHa2	Adipocyte	Fat body	L/A	IPC regulation
ETH	Inka cells	Trachea	L/P/A	Ecdysis, JH production, reproduction, courtship memory
Brain neurons that partake in orchestration				
SIFa	SIFan	PI	L/A	Feeding, mating, sleep
LK	LHLK	Brain	L/A	osmotic homeostasis, metabolism, sleep, thirst-related learning
PDF	PDFtri	Tritoc.	A^5^	Adult eclosion?
PDF, sNPF	sLNv	Clock	L/A^6^	Clock neuromodulator, activity, sleep
TK, MIP	ICN	Brain	L/A	IPC regulation, growth
AstA	AstAn, PLP	Brain	A	Feeding, sleep
MIP	MIPn	Brain	A	Feeding, olfaction, sleep, reproduction
Thoracico-abdominal ganglion neurosecretory cells^7^				
dFMRFa	Tv	T1-T3	L/A	Locomotion, flight
CAPA	Va	AbdG	L/A	Osmotic homeostasis, chill coma recovery
LK, DH44	ABLK	AbdG	L/A	Osmotic homeostasis, tracheal air-filling (at ecdysis)
Burs α	–	AbdG	L/A	Cuticle tanning, wing expansion
GPB 5	–	AbdG	L/A	Osmotic homeostasis?
PDF	–	AbdG	L/A	Motility renal tubules^8^, defecation in larvae
DH31	–	AbdG	L/A	Osmotic homeostasis?
Orcokinin A	–	AbdG	L/A	?
Thoracico-abdominal ganglion efferent neurons (could be neurosecretory)				
DILP7	dMP2	AbdG	L/A	Gut function, reproduction
ITP	iag	AbdG	L/A	Osmotic homeostasis?
Luqin (RYamide)	–	AbdG	L/A	Osmotic homeostasis?
Proctolin		AbdG		Gut contraction?
AstA		AbdG		Gut contraction?
Midgut enteroendocrine cells (EECs)^9^				
AstA	EECs P	Midgut	L/A	Signal to AKH cells and IPCs?
AstB/MIP	EECs M, P	Midgut	L/A	?
AstC	EECs A, M, P	Midgut	L/A	?
Bursicon α	EECs P	Midgut	A	Signal to AKH cells (indirect)
CCHa1	EECs P	Midgut	L/A	?
CCHa2	EECs A, P	Midgut	L/A	Signal to IPCs
DH31	EECs P	Midgut	L/A	Signal to R tubules, gut
NPF	EECs A, M	Midgut	L/A	Signal to R tubules, gut
Orcokinin B	EECs M	Midgut	L/A	?
TK	EECs A, M, P^10^	Midgut	L/A	Lipid metabolism in gut

Acronyms for peptides are given in Table 1. References are given in the text of the review. The regions are as follows: *PL*, pars lateralis; *PI*, pars intercerebralis; *SEZ*, subesophageal zone; *Tritoc.*, tritocerebrum; *T1-T3*, thoracic neuromeres 1–3; *AbdG*, abdominal ganglion

^1^L, larval; A, adult

^2^DILP1 not present in larvae (only in pupae and early adult)

^3^Also designated DH44n

^4^No TK in larvae

^5^Undergo apoptosis in early adult (after adult eclosion)

^6^No sNPF in larval neurons

^7^All functions only shown for adults

^8^Leads to increased secretion in tubules

^9^A, anterior; M, median; and P, posterior midgut

^10^Also in anterior hindgut

## MNCs: regulation of insulin-producing cells in *Drosophila* and interorgan communication

There are 14 IPCs located in the pars intercerebralis (MNC group) and it has been assumed that these cells function as a group, i.e., that regulatory factors act on all IPCs. However, it seems that there are three subtypes of IPCs with respect to nutrient sensing: (1) IPCs with autonomous glucose sensing, (2) IPCs without glucose sensing and (3) those that receive inputs from two glucose-sensing DLPs (designated CNs) (Oh et al. 2019). Another heterogeneity is that an IPC subpopulation co-expresses DSK (Söderberg et al. 2012). The exact sites where specific regulatory inputs to the IPCs appear has not been established, although some connections between IPCs and other neurons have been analyzed in the first instar larvae of *Drosophila* (Schlegel et al. 2016). Based on anatomy and dendritic markers, it is likely that inputs to IPCs are on two sets of arborizations in the pars intercerebralis and one set in tritocerebrum (see Fig. 3a). The two sets of IPC “dendrites,” longer and shorter, are seen in Fig. 3(a’, e) (see also Supplementary Fig. 1).

**Fig. 4:**
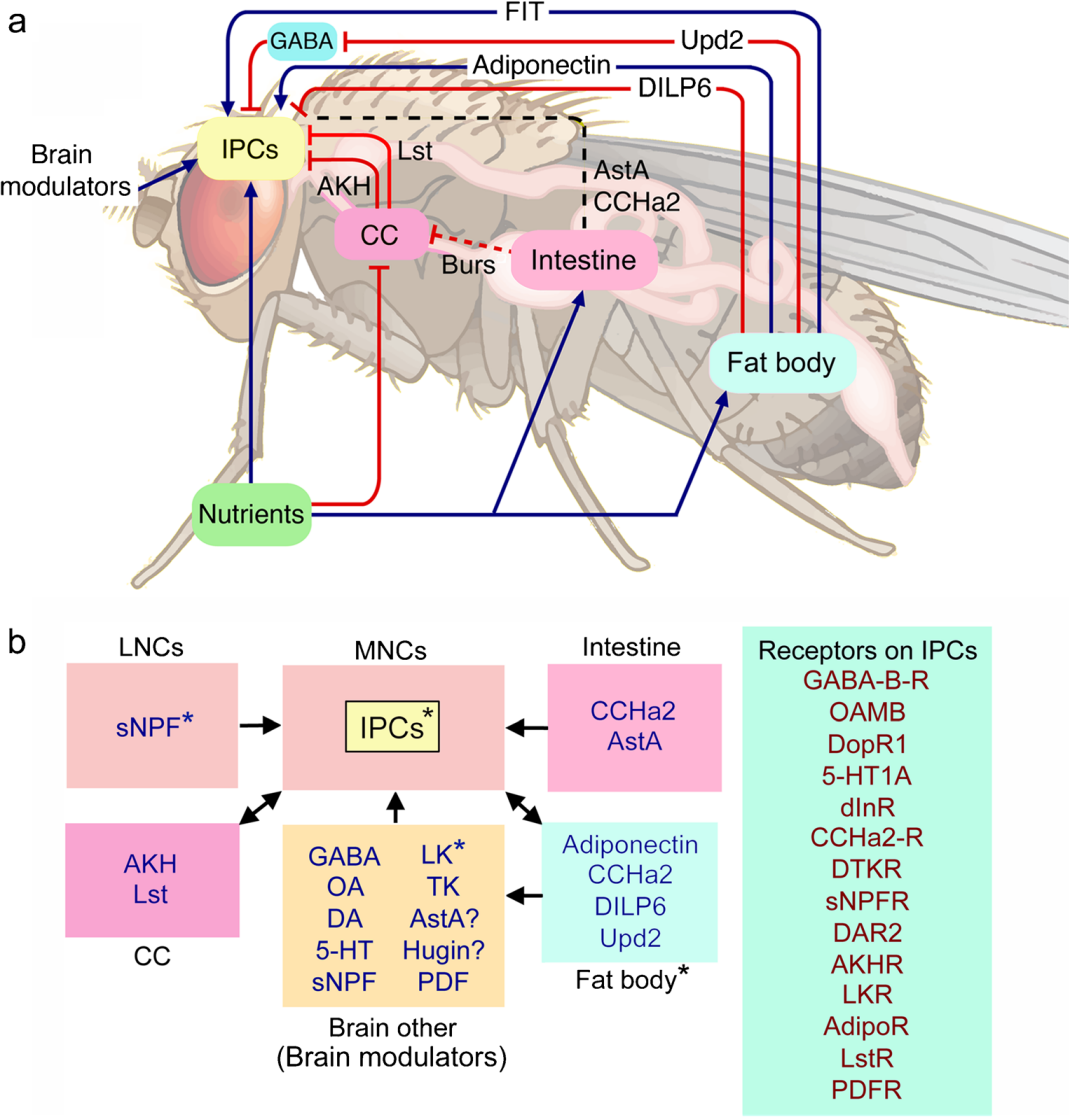
Interorgan communication: regulation of insulin-producing cells (IPCs) in Drosophila. **a** Scheme with factors that regulate insulin-producing cells (IPCs) in the adult brain of *Drosophila*. Blue arrows depict stimulatory inputs and red bars show inhibitory ones. Dashed black line indicates incompletely known mechanisms. The IPCs are also regulated by neurons in the brain (brain modulators; see panel **b** for details). The fat body is nutrient sensing and releases adiponectin-like polypeptide, Upd2 (unpaired-2), and DILP6 after carbohydrate intake. Adiponectin and DILP6 act directly on the IPCs. Upd2 acts (inhibitory) on GABAergic brain neurons and thereby lifts inhibition of the IPCs. Another factor FIT (female-specific independent of transformer) is a signal released from the fat body after a protein meal. The corpora cardiaca (CC), under conditions of low sugar, releases limostatin (Lst) and adipokinetic hormone (AKH) and thereby inhibits release of DILPs. The intestine has nutrient-sensing enteroendocrine cells and there is release of at least some peptide hormones into the circulation. Two gut peptides have been shown to act on IPCs, allatostatin A (AstA), and CCHamide2 (CCHa2), whereas bursicon (Burs) from the intestine acts on brain neurons, which in turn act on CC to diminish AKH production (dashed line indicates indirect action via brain). Acronyms or peptides are given in Table 1. For references to the original data, see the text. This figure is slightly modified from Nässel and Zandawala et al. (2019) with permission. **b** Block diagram of factors acting on IPCs and receptors expressed on these cells. The IPCs are nutrient sensing, as are peptidergic cells in the brain and fat body (asterisks). Insulin-like peptides (DILPs) released from IPCs act on CC and fat body and regulate AKH and DILP6 release, respectively. Receptor acronyms: GABA-B-R, metabotropic GABA receptor; OAMB, octopamine receptor (mushroom body); DopR1, dopamine receptor 1; 5-HT1A, serotonin receptor 1A; dInR, insulin receptor; DTKR, TK receptor; DAR2, AstA receptor 2; AdipoR, adiponectin receptor

As mentioned above, some of the IPCs in adult flies are intrinsically nutrient sensing (glucose, amino acids) and they additionally receive regulatory signals from brain neurons, CC, the intestine and the fat body (Fig. 4a) (Ahmad et al. 2019; Nässel and Vanden Broeck 2016; Nässel and Zandawala 2019; Oh et al. 2019). The brain neuromodulators and other factors are shown in Fig. 4(b) and this figure also lists the different receptors expressed by the IPCs. It is apparent that the IPCs receive an impressive amount of regulatory inputs with at least 14 receptors expressed in the adult fly (Ahmad et al. 2019; Nässel et al. 2015; Nässel and Vanden Broeck 2016; Nässel and Zandawala 2019). These factors include GABA, serotonin, dopamine, octopamine and several neuropeptides. There are additional factors regulating IPCs in the larva: AKH, Dawdle (Daw), Glass bottom boat, stunted and Eiger, growth-blocking peptide (Agrawal et al. 2016; Ballard et al. 2010; Delanoue et al. 2016; Ghosh and O'Connor 2014; Kim and Neufeld 2015; Koyama and Mirth 2016). In addition, 20-hydroxyecdysone (20E) acts on IPCs to regulate DILP production (Buhler et al. 2018). The factors from the fat body and intestine are released in response to nutrients such as dietary sugar, proteins (amino acids) and lipids (Ahmad et al. 2019; Owusu-Ansah and Perrimon 2014).

**Fig. 5:**
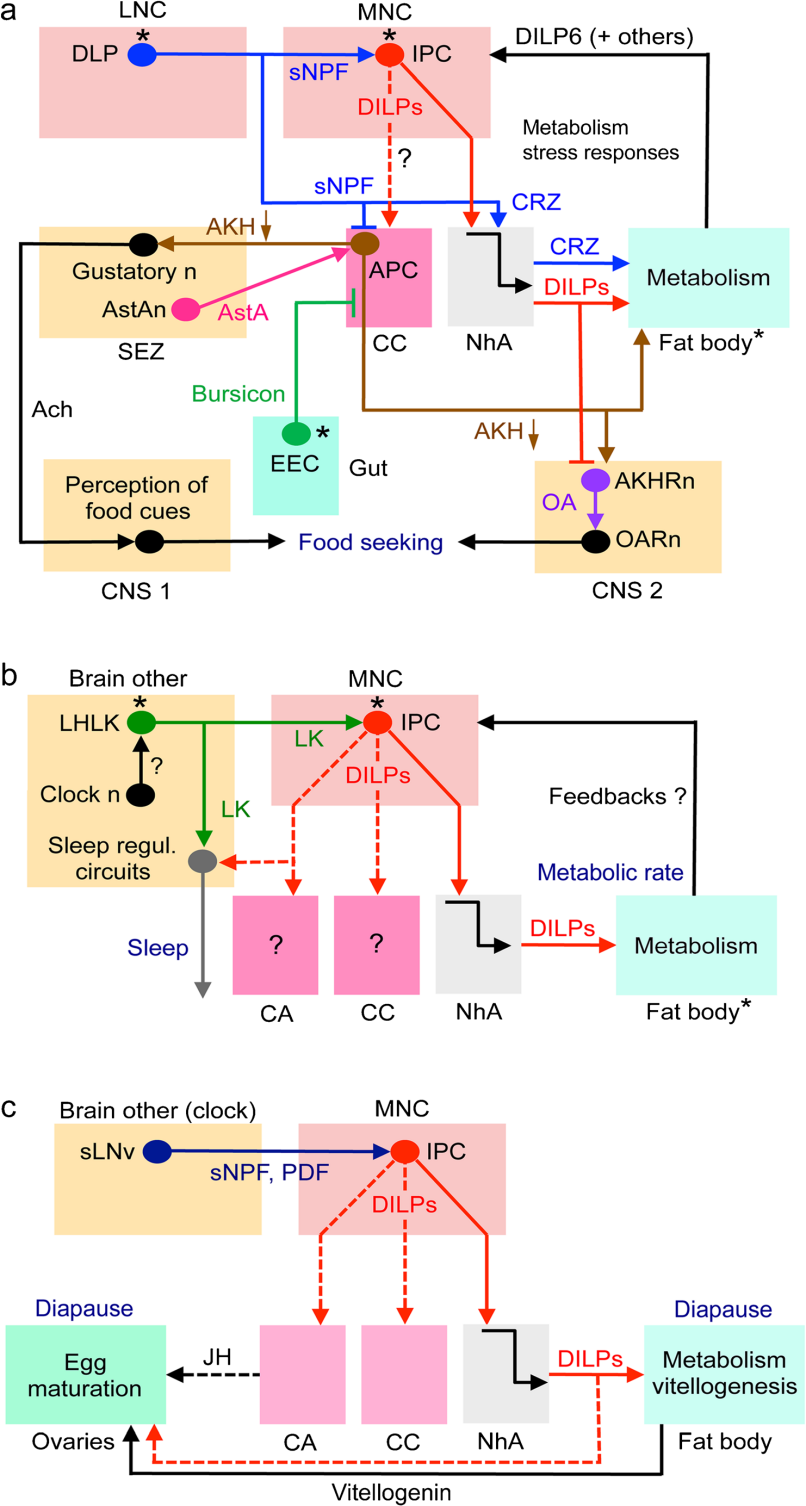
Schemes depicting hormonal axes involving insulin-producing cells (IPCs) in adult flies. Note that in this and the following figures with schematic circuits/axes, only one cell of each type is depicted for simplicity. **a** Adult DLP (LNC) pathway with sNPF and corazonin (CRZ) and regulation of glucose homeostasis, food search and stress responses via IPCs and AKH-producing cells (APCs). Asterisks indicate neurons/cells that are cell autonomously nutrient sensing. The DLPs in the LNC group produce CRZ and sNPF and supply axon terminations to IPCs and APCs. The DLP-derived sNPF regulates IPCs and APCs in corpora cardiaca (CC) and thereby affects glucose homeostasis and metabolic stress responses (Kapan et al. 2012; Oh et al. 2019), whereas CRZ is released into the circulation from the neurohemal area (NhA) associated with CC, the foregut and anterior aorta (Kubrak et al. 2016). CRZ acts on the fat body to regulate metabolic stress and homeostasis (Kubrak et al. 2016). sNPF activates IPCs to increase DILP release and inhibits the APCs to decrease AKH release and thereby affects carbohydrate homeostasis (Kapan et al. 2012; Oh et al. 2019). The signaling from IPCs to APCs in CC (dashed arrow) specifically in response to sNPF has not been demonstrated but DILPs do act on APCs, at least in larvae (Bader et al. 2013). AKH is also known to regulate the sensitivity of AKH receptor–expressing sweet-sensing gustatory neurons that mediate sweet taste (Bharucha et al. 2008; Jourjine et al. 2016). Note that it is not clear whether AKH acts on gustatory neuron processes within the SEZ or their cell body/dendrites in the periphery. Enteroendocrine cells (EECs) of the midgut release bursicon that indirectly inhibits APCs in CC resulting in decreased release of AKH and thus affect glucose homeostasis (Scopelliti et al. 2014). Another regulator of IPCs and APCs is allatostatin A (AstA) but the neuronal pathway mediating this has not been clearly shown (Hentze et al. 2015). Thus, it might be that instead of SEZ neurons, AstA is derived from gut EECs. The fat body may feed back to IPCs by means of DILP6 and other factors such as Upd2, adiponectin and CCHa2 (see Fig. 6, Ahmad et al. 2019; Nässel and Zandawala 2019). Finally, DILPs and AKH regulate octopamine (OA)-producing neurons (AKHRn) that express AKH and DILP receptors. These AKHRn in turn act on octopamine receptor–expressing neurons (OARn) to activate locomotion (Yu et al. 2016). Thus, AKH regulates sensitivity of taste neurons and activates locomotion to increase food search. AKH action also encompasses regulation of activity/rest, depending on time of day (Pauls et al. 2020). Ach, acetylcholine; EEC, enteroendocrine cell; **b** the IPCs are regulated by leucokinin (LK)-producing neurons, LHLK, in the lateral horn of the brain. The LHLKs, which are under influence of clock neurons (Cavey et al. 2016), also mediate starvation-dependent changes in sleep (Yurgel et al. 2019). The nutrient-sensing LHLKs are part of an LK system in the brain and ventral nerve cord that also regulates physiological processes such as diuresis, metabolism, and organismal activity (Zandawala et al. 2018b). **c** Activation of IPCs blocks reproductive diapause (i.e., blocks ovarian arrest). The clock neurons, sLNv, use sNPF and PDF to activate the IPCs, which leads to inhibition of diapause, likely due to DILP-mediated activation of vitellogenesis in fat body and egg maturation in ovaries (Nagy et al. 2019b). This probably also involves DILP stimulation of corpora allata (CA) and production of juvenile hormone (JH). Inputs to IPCs from another set of clock neurons (DN1s; not shown here) were shown in another study to regulate feeding rhythm and metabolism (Barber et al. 2016)

In adults, the IPCs are central to the regulation of metabolism, appetite and food seeking as shown in Fig. 5(a). LNCs (DLPs) use sNPF to activate IPCs and inhibit AKH-producing cells (APCs) in CC and CRZ to signal systemically to the fat body (Kapan et al. 2012; Kubrak et al. 2016; Oh et al. 2019). The altered DILP and AKH signaling affects metabolism, gustatory sensitivity and circuits that regulate food seeking (Bharucha et al. 2008; Jourjine et al. 2016; Yu et al. 2016). Feedback to IPCs and APCs is derived from the fat body and enteroendocrine cells (EECs) of the intestine, respectively (see Ahmad et al. 2019; Nässel and Vanden Broeck 2016; Scopelliti et al. 2014). This is an example where a set of LNCs, part of which are nutrient sensing (Miyamoto et al. 2012; Oh et al. 2019), activates MNCs and regulate food seeking, feeding and metabolism. Since both LNCs and MNCs (i.e., DLPs and IPCs) display autonomous nutrient sensing, the final output from the IPCs is the sum of the inputs from DLPs and their own sensing.

Another pathway that involves IPCs regulates metabolism-sleep interactions (Fig. 5b). A pair of brain interneurons (LHLK) producing the neuropeptide leucokinin (LK) acts on IPCs and other neuronal circuits in the brain regulating sleep. The LHLKs are glucose sensing and this LK signaling requires LK receptor expression on IPCs and affects starvation-mediated changes in sleep (Yurgel et al. 2019). The LHLKs are in turn regulated by clock neurons (Cavey et al. 2016). IPCs and specifically DILP2, are also implicated in compensatory regulation of sleep rebound after starvation-induced sleep deprivation (Brown et al. 2020). Furthermore, the nutrient-sensing LHLKs are part of an LK system in the brain and ventral nerve cord (totally about 24 LK neurons) that together regulate physiological processes such as diuresis, feeding, metabolism and organismal sleep activity (Yurgel et al. 2019; Zandawala et al. 2018b). It can be added that the LHLKs also partake in a memory circuit that involves dopaminergic neurons and mushroom body (MB) neurons (Senapati et al. 2019). Thirst and hunger signals to LHLKs are relayed to dopaminergic neurons that in turn affect expression of water-related memory and sugar-related memory in the MBs. In the same circuit, neurons producing serotonin and NPF relay hunger signals to specific dopaminergic inputs to the MBs (Senapati et al. 2019). Thus, LHLKs play a critical role in several aspects of daily physiology of flies, relaying hunger and thirst signals to affect metabolism, sleep and memory formation and do so by interacting with IPCs and MBs.

A third pathway involving IPCs regulates ovary and egg maturation (Fig. 5c). *Drosophila* can enter adult reproductive diapause when environmental conditions are adverse (Kubrak et al. 2014; Saunders et al. 1989; Tatar and Yin 2001). Activation of IPCs by sNPF from clock neurons blocks reproductive diapause in the fly (Fig. 5c) (Nagy et al. 2019a). This block is likely to result from decreased production of vitellogenin from fat body, altered metabolism and hormonal effects on ovary maturation. These could be direct effects of IIS on ovaries and/or indirect via juvenile hormone (JH) from CA. It has also been shown that IIS is required for upregulating the female remating rate after the post-mating refractory period in *Drosophila* (Wigby et al. 2011). Therefore, it seems that the nutrient-responsive IIS pathway is critical for regulating female mating behavior in response to availability of relevant food sources. In summary, at least three different hormonal axes involving IPCs and DILPs have been described that regulate (1) appetite, feeding and metabolism; (2) sleep metabolism interactions; and (3) mating, reproduction and diapause.

## MNCs: functions of DH44-producing neurons in *Drosophila*

**Fig. 6:**
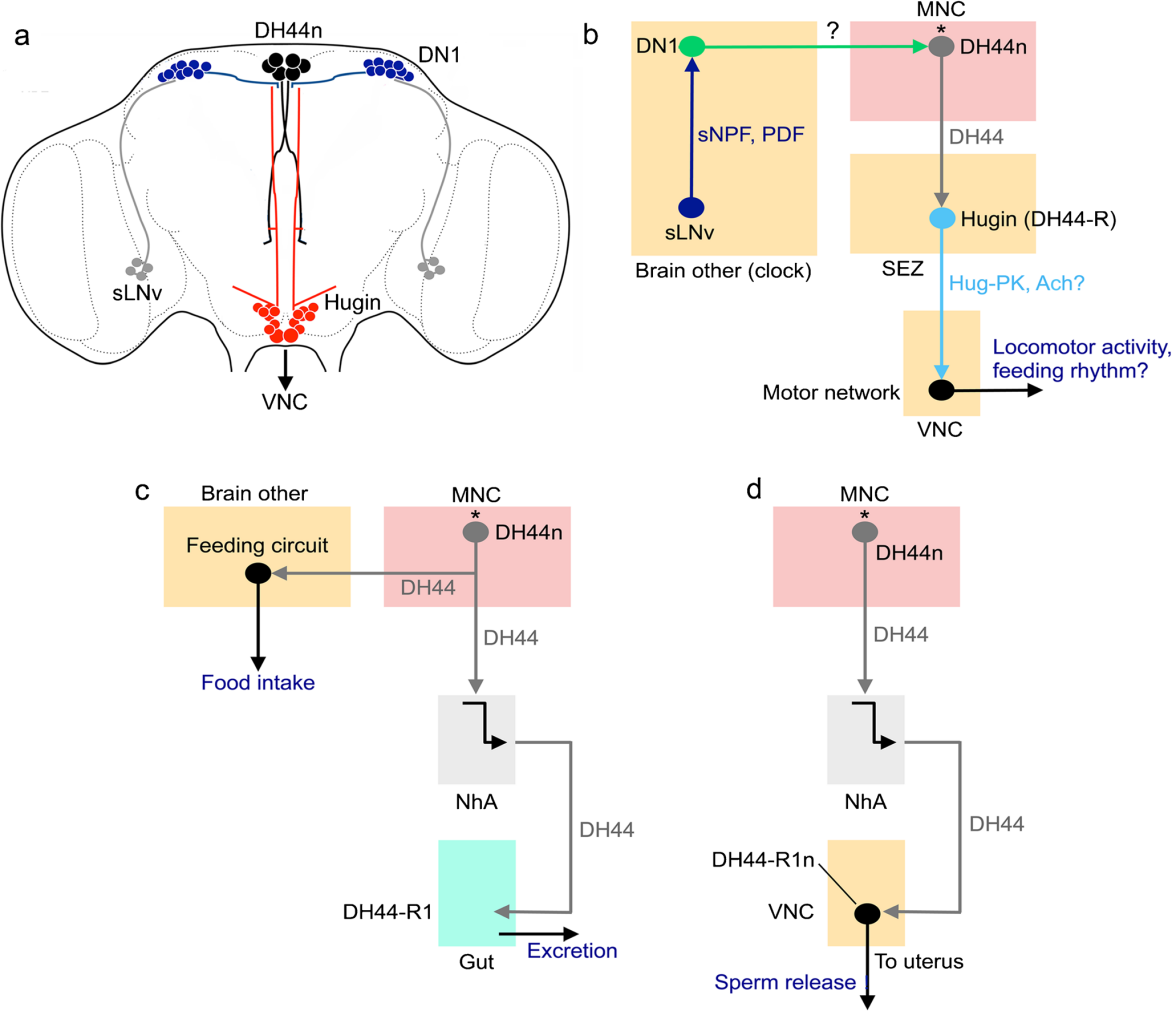
Schemes depicting hormonal axes involving DH44-producing median neurosecretory cells (DH44n) in adult flies. **a** Localization of DH44 neurons (DH44n), Hugin cells, and the clock neurons DN1 and sLNv in the adult fly brain. The sLNv produce sNPF and PDF and the DN1 are heterogeneous and produce a variety of neuropeptides (see Abruzzi et al. 2017). **b** Block diagram depicting interactions between the neurons shown in panel **a**. The sLNv neurons act (via either sNPF or PDF) on DN1s to activate (substance not known) DH44 neurons that in turn act on a subset of DH44 receptor–expressing (DH44-R) Hugin cells (King et al. 2017). The Hugin cells utilize Hugin-pyrokinin (Hug-PK) and/or acetylcholine (Ach) to activate motor neuron circuits in the ventral nerve cord (VNC). This pathway regulates locomotor activity and in conjunction with SIFamide neurons also modulates feeding rhythms (Dreyer et al. 2019; King et al. 2017). Note that the DH44n (asterisk) are amino acid and glucose sensing (Dus et al. 2015; Yang et al. 2018). Novel data show that the Hugin cells receive inputs from sleep-promoting neurons of dorsal fan-shaped body (dFB) and that Hugin cells also act on sLNvs (not shown in diagram) (King et al. 2020). This circuit links homeostatic sleep drive and the circadian system. **c** The nutrient-sensing DH44 neurons induce feeding and defecation (excretion) (Dus et al. 2015). Hormonally released DH44 acts on DH44-R1-expressing muscles in the intestine to induce excretion. The DH44n also act on circuits in the brain regulating feeding. **d** In female flies, the DH44n release DH44 that acts on DH44-R1-expressing efferent neurons that innervate the uterus and thereby induces sperm release from spermatheca (Lee et al. 2015)

DH44 was first identified as hormone regulating secretion in Malpighian tubules (Cabrero et al. 2002; Kataoka et al. 1989b) but, as we will show here, it is now known that it plays a multitude of roles in *Drosophila* physiology (see Nässel and Zandawala 2019). There are six MNCs that express DH44 and their morphology resembles that of IPCs (Figs. 3g and 6a). The six DH44 neurons are amino acid and glucose sensing (Dus et al. 2015; Yang et al. 2018). In the adult fly, these six MNCs also express DILP2 (Ohhara et al. 2018) but the functional role of DILP in these cells is unclear.

The DH44 neurons appear to be part of the output from the circadian clock in *Drosophila* (Fig. 6a, b). These neurons receive inputs from DN1 clock neurons (transmitter unknown), which in turn are activated by sLNv clock neurons that use either sNPF or PDF as neuromodulators (King et al. 2017). The DH44 neurons activate DH44 receptor 1 on peptidergic Hugin neurons in the subesophageal zone (SEZ) that in turn have connections to motor circuits in the ventral nerve cord that drive locomotor activity (Fig. 6a, b). This pathway in conjunction with the profusely arborizing SIFamide neurons also modulates feeding rhythms (Dreyer et al. 2019; King et al. 2017) (Fig. 6b).

Furthermore, the nutrient-sensing DH44 neurons stimulate feeding and defecation (excretion) (Dus et al. 2015; Yang et al. 2018) as shown in Fig. 6(c). The DH44 neurons act on circuits in the brain regulating feeding and hormonally released DH44 acts on DH44-R1-expressing muscles in the intestine to induce excretion. Finally, in female flies, it has been shown that the DH44 neurons release DH44 that acts on DH44-R1-expressing efferent neurons in abdominal ganglia that innervate the uterus and thereby triggers sperm release from spermatheca (Lee et al. 2015) (Fig. 6d).

In summary, the DH44-expressing MNCs regulate (1) locomotor activity and feeding rhythms, (2) feeding and excretion and (3) sperm release from spermatheca. It is not clear whether these represent three different axes or if there is an interactive link between these functional pathways.

## Diverse hormonal and peptidergic systems regulate feeding and other associated behaviors

**Fig. 7:**
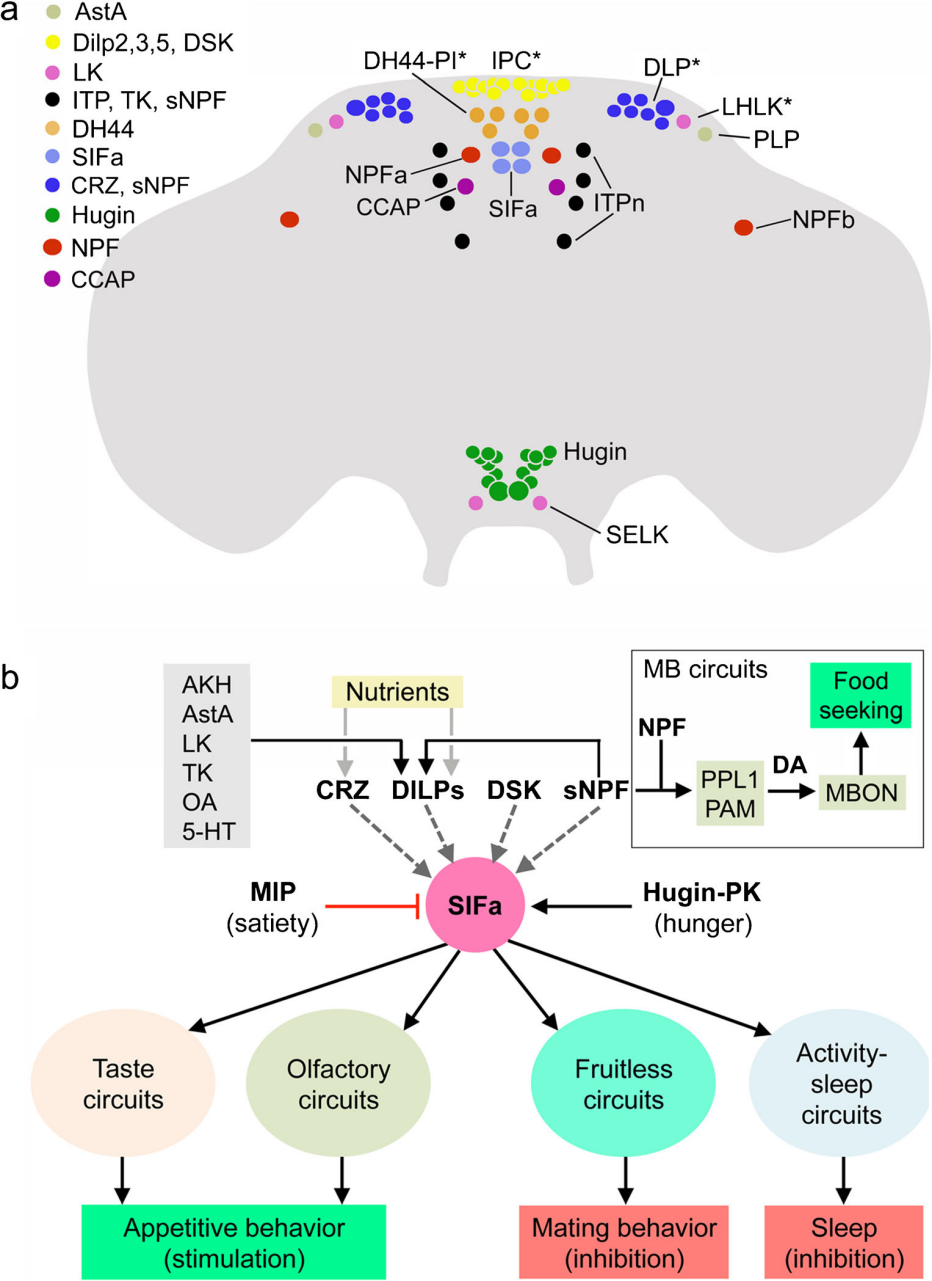
Schemes depicting hormonal systems that regulate feeding and associated behaviors. **a** Distribution of cell bodies of peptidergic neuroendocrine cells in the brain of *Drosophila* that play roles in feeding. These are neurosecretory cells in MNC (IPC and DH44-PI) and LNC groups (ITPn and DLP) and interneurons located in distinct brain regions (LHLK, PLP, NPF, SIFa, Hugin and SELK); a few of the Hugin cells are neurosecretory cells. The neuron groups indicated with asterisks are nutrient sensing (only a subset of the DLPs) and the Hugin cells in the subesophageal zone receive gustatory inputs. The peptides released from these cells are shown in the legend in the upper left part of the figure (acronyms as in Table 1). Note that also circuits associated with the mushroom bodies (see box in panel **b**) are linked to some of the peptidergic systems shown and are involved in regulation of food seeking and feeding (Tsao et al. 2018). See text for literature references. **b** Four neurons producing SIFamide (SIFa) have arborizations that are widely spread throughout the brain. These SIFa neurons coordinate appetitive behavior but also influence mating and sleep (Dreyer et al. 2019; Martelli et al. 2017; Terhzaz et al. 2007). The SIFa neurons are under direct regulation by peptidergic satiety inputs (myoinhibitory peptide, MIP) and hunger inputs (Hugin-PK). SIFa neurons act on gustatory and olfactory sensory neurons, as well as sets of neurons expressing the transcription factor Fruitless, known to regulate sex-specific behavior. They also act on MNCs in the pars intercerebralis that signal with diuretic hormone 44 and insulins (not shown in the figure), as well as specific neuronal circuits regulating sleep. Anatomical studies (reconstitution of split-GFP) suggest that the SIFa neurons also receive inputs from neurons that play roles in feeding and metabolism that produce corazonin (CRZ), DILPs, sulfakinin (DSK) and sNPF (Martelli et al. 2017). The IPCs are in turn regulated by sNPF and several other factors shown in the gray box in the upper left. Mushroom body–associated circuits (in box) are also involved in the regulation of food seeking; sNPF and NPF act on different sets of dopaminergic (DA) neurons (PPL, PAM), which in turn act on specific sets of mushroom body output neurons (MBONs) that induce food-seeking behavior (Tsao et al. 2018). This figure is updated from Nässel et al. (2019), which was based on data from Martelli et al. (2017) and Tsao et al. (2018)

Regulation of feeding is complex in *Drosophila* and relies on several sets of peptidergic neurons spread out in the brain and SEZ and is not exclusively associated with the LNC-MNC cell groups (Lin et al. 2019; Nagata and Zhou 2019; Nässel and Zandawala 2019). The major sets of peptidergic neurons involved in feeding are shown in Fig. 7(a). These produce the peptides allatostatin A (AstA), crustacean cardioactive peptide (CCAP), CRZ, DILPs, DH44, Hugin-PK, ITP, LK, NPF, SIFa, sNPF and TK. A few of these are neurosecretory cells in the MNC (IPC and DH44-PI) and LNC groups (ITPn and DLP) but several other neurons are located in distinct brain regions (LHLK, PLP, NPFa, NPFb, SIFa, Hugin and SELK neurons); most are interneurons but a few of the Hugin cells are neurosecretory cells. Some of these neurons are autonomously nutrient sensing (DH44-PI, IPCs, DLPs and LHLKs; asterisks in Fig. 7a) (Dus et al. 2015; Kreneisz et al. 2010; Miyamoto et al. 2012; Park et al. 2014; Yurgel et al. 2019) and some receive inputs from gustatory neurons (Hugin cells) (Melcher and Pankratz 2005). Interactions between a few of these neurons have been described: LHLKs and DLPs provide inputs to IPCs, using sNPF and LK, respectively (Kapan et al. 2012; Yurgel et al. 2019; Zandawala et al. 2018b); SIFa neurons receive inputs from Hugin cells (Hug-PK), MIP-expressing neurons (MIP) and probably IPCs (DILPs) and DLPs (CRZ, sNPF) (Martelli et al. 2017). Finally, IPCs seem to signal to ITPn, Hugin cells and AKH-producing cells in CC in an Imp-L2- and dInR-dependent fashion (see Fig. 3a; Bader et al. 2013) but the function of these interactions is not yet known.

Since regulation of feeding in *Drosophila* and other insects has been extensively reviewed recently (Audsley and Weaver 2009; Lin et al. 2019; Nagata and Zhou 2019; Nässel and Zandawala 2019; Pool and Scott 2014), we will just make some remarks here that refer to Fig. 7. A few sets of neurons appear central in regulation of appetite, satiety and feeding (and metabolism). Some of these are the neurosecretory cells (see Fig. 7) such as IPCs that regulate metabolism, appetite and satiety with DILPs and DSK (Broughton et al. 2005; Kim et al. 2017; Nässel et al. 2013; Root et al. 2011; Semaniuk et al. 2018; Söderberg et al. 2012; Yu et al. 2016); DH44-PI that stimulate food intake with DH44 (Dus et al. 2015; Yang et al. 2018); DLPs that use CRZ to regulate metabolism and indirect feeding (Kubrak et al. 2016); and ITPn that suppress feeding through ITP (Galikova et al. 2018). The endocrine cells of CC produce AKH and limostatin to modulate gustation (stimulate sweet and inhibit bitter) and stimulate appetite and feeding (Bharucha et al. 2008; Inagaki et al. 2014; Jourjine et al. 2016; Lee and Park 2004; Yu et al. 2016).

Other peptidergic signals involved in feeding and metabolism are mediated by different interneurons distributed in different brain areas (Fig. 7a). These interneuronal peptides are AstA, CCAP, Hugin-PK, LK, NPF and SIFa. It is possible that also other interneurons that are not shown in Fig. 7(a) utilize peptides, such as DH44, DSK, NPF, sNPF and TK to regulate feeding. AstA regulates IPCs, inhibits feeding and promotes sleep (Chen et al. 2016b; Hentze et al. 2015; Hergarden et al. 2012; Wang et al. 2012a). The two CCAP neurons regulate a set of two NPF neurons (NPFa) and thus modulate sugar preference and stimulate feeding (Williams et al. 2020). In larvae, Hugin neurons receive inputs from gustatory neurons and inhibit feeding (Melcher and Pankratz 2005); in adults, these neurons relay hunger signals to SIFa neurons and thereby affect appetite and feeding (Martelli et al. 2017). LHLK neurons use LK to regulate IPCs but also other neuronal circuits to regulate metabolism and sleep, as well as water- and sugar-associated memory formation (Senapati et al. 2019; Yurgel et al. 2019; Zandawala et al. 2018b). NPF neurons have been extensively studied in stimulation of larval feeding (Shen and Cai 2001; Wang et al. 2013; Wu et al. 2005) but these neurons are also important in adult feeding (Chung et al. 2017; Pu et al. 2018; Tsao et al. 2018; Williams et al. 2020). SIFa neurons are central in balancing feeding, sleep and reproductive behavior (Dreyer et al. 2019; Martelli et al. 2017; Terhzaz et al. 2007) and will be dealt with in more detail below. Unspecified neurons producing sNPF are known to regulate feeding in larvae (Lee et al. 2004), as well as in adults (Tsao et al. 2018), where food search is also regulated via sNPF modulation of olfactory circuits (Root et al. 2011). To summarize, several sets of interneurons/neurosecretory cells and peptides regulate appetite and feeding; however, only in a few cases are the interactions between these different neurons known. These will be shown next.

**Fig. 8:**
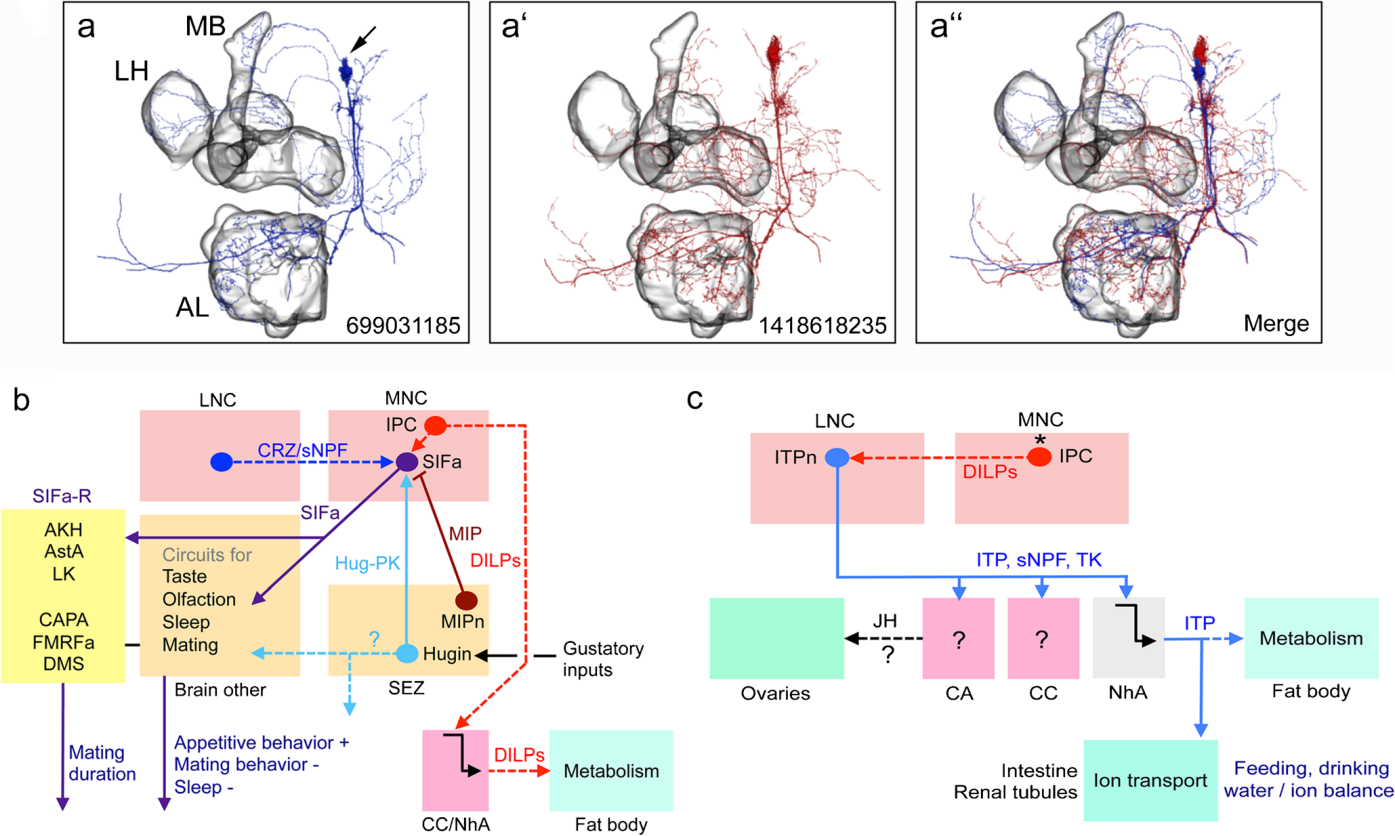
Schemes depicting circuits/axes involving SIFamide- and ion transport peptide-producing interneurons (ITPn) in adult flies. (a, a’) Reconstructions of two SIFa neurons from serial electron microscopic sections (combined in the third panel, a”). MB, mushroom body, LH, lateral horn, AL, antennal lobe. The numbers identify the neurons in the database. These panels were compiled from data in neuPRINT (https://neuprint.janelia.org) (Clements et al. 2020; Xu et al. 2020; Zheng et al. 2018). (b) SIFamide (SIFa)-producing interneurons are central in regulating appetitive behavior and decreasing sleep and mating behavior. As also shown in Fig. 7, the SIFa neurons are modulated by neurons producing MIP, Hugin-PK and possibly CRZ/sNPF. The SIFa neurons also target SIFa receptor–expressing peptidergic neurons (SIFaR) in the CNS. Of these, AKH-, AstA- and LK-expressing neurons induce shorter mating duration and CAPA-, FMRFa- and DMS-expressing ones induce longer mating (Wong et al. 2019). Dashed lines indicate that actions have not been shown experimentally in the context of SIFa signaling. Peptide acronyms are as in Table 1. (c) Ion transport peptide (ITP) is produced by a set of LNCs (ITPn) that has axon terminations in corpora cardiaca (CC) and allata (CA), as well as neurohemal areas (NhA). ITP regulates water intake and water reabsorption/excretion and possibly metabolism (Galikova et al. 2018). The ITPn are targets of DILP2 from the IPCs (Bader et al. 2013) and express the LK receptor (Zandawala et al. 2018b) but the functional aspects of this are not known

As an example of interactions between neurons regulating feeding, we show the inputs and outputs of SIFa neurons (Figs. 7b and 8a). The four neurons producing SIFa have arborizations that are spread throughout the brain and coordinate appetitive behavior but also inhibit mating and sleep (Dreyer et al. 2019; Martelli et al. 2017; Terhzaz et al. 2007). The SIFa neurons are regulated by peptidergic satiety inputs (MIP) and hunger inputs (Hugin-PK). In turn, the SIFa neurons act on gustatory and olfactory sensory neurons, as well as sets of neurons expressing the transcription factor Fruitless that regulate sex-specific behavior. Furthermore, they act on MNCs in the pars intercerebralis that signal with DH44 and DILPs, as well as specific neuronal circuits regulating sleep. The SIFa neurons may also receive inputs from neurons that play roles in feeding and metabolism that produce CRZ, DILPs, DSK and sNPF (Martelli et al. 2017). Thus, the SIFa neurons seem to be at the center of sensing nutrient status to balance opposing behaviors: appetitive behavior versus mating and sleep (Dreyer et al. 2019; Martelli et al. 2017).

A set of neurons (ITPn) producing ITP have recently been shown to play a central role in regulation of feeding, drinking and excretion (Galikova et al. 2018). The ITPn are LNCs and are likely to act by systemic release of ITP, possibly acting on the hindgut, Malpighian tubules and fat body (Fig. 8b). Indirect evidence suggests that the IPCs signal to the ITPn in an Imp-L2- and dInR-dependent fashion (Bader et al. 2013). The ITPn also produce sNPF and TK and knockdown of these peptides in ITPn increased the sensitivity to starvation and desiccation (Kahsai et al. 2010) indicating that the ITPn are important for metabolism and water homeostasis.

## Neurosecretory systems in the ventral nerve cord

**Fig. 9:**
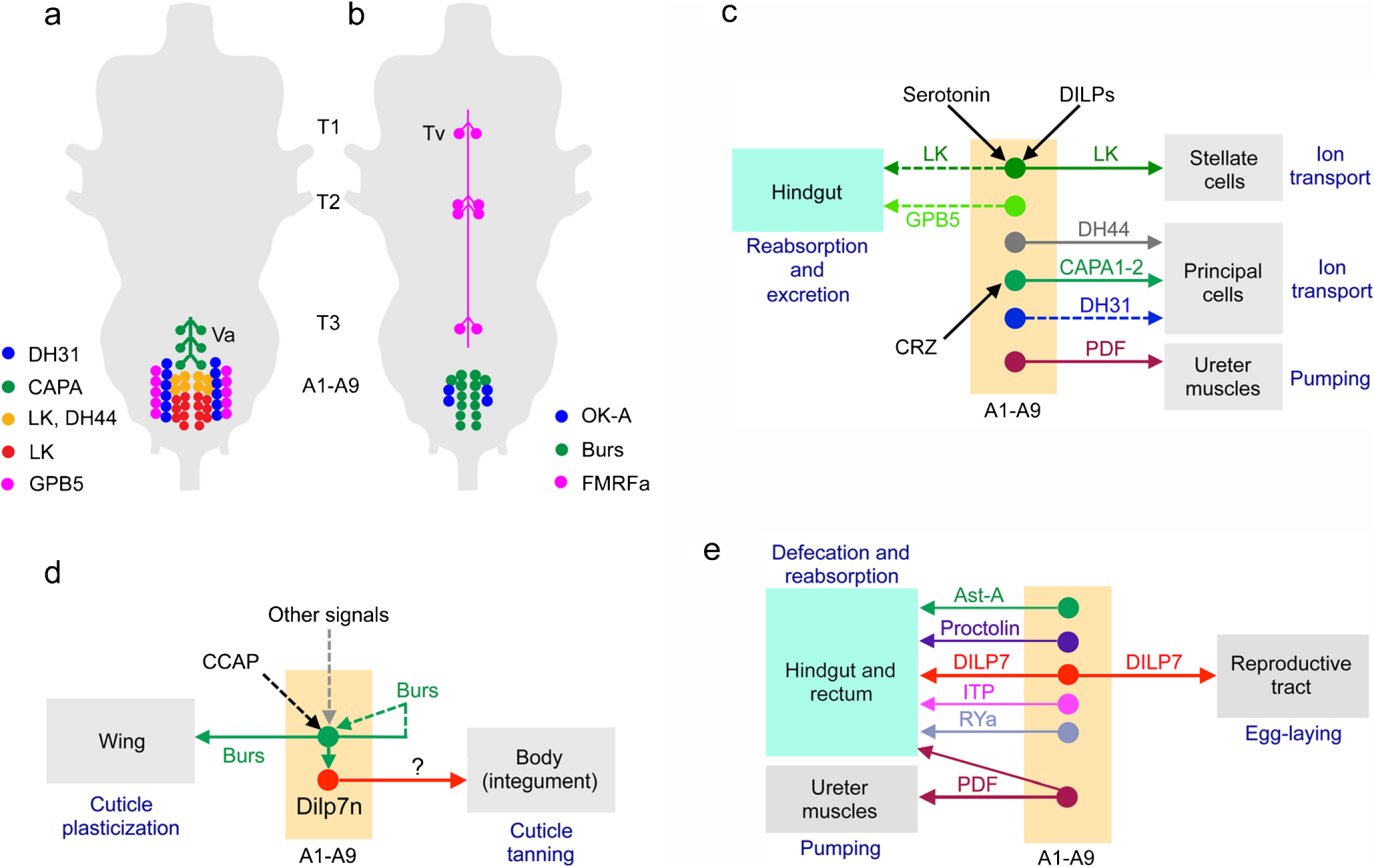
Schemes depicting neurosecretory and efferent neuronal systems in the adult ventral nerve cord. **a**, **b** Cell bodies of neurosecretory cells in the adult ventral nerve cord (VNC) are mainly found in abdominal neuromeres and only a set of FMRFamide-expressing cells are known in the thoracic neuromeres. The peptide acronyms are explained in Table 1. **a** Peptide hormones regulating water and ion balance, as well as stress responses. The Va neurons have axon terminations in a neurohemal area in the dorsal neural sheath of the VNC, the others terminate on muscles in the body wall. **b** Peptides with unclear functions in the adult. The Tv cells have axon terminations in a plexus forming a neurohemal area in the dorsal neural sheath of the VNC, the others terminate on muscles in the body wall. **c** Peptide hormones in abdominal neuromeres that regulate water and ion balance. Dashed lines indicate that actions from abdominal cells have not been shown experimentally. Some of the cells are regulated by specific substances (serotonin, DILPs and CRZ); the LK producing ABLK neurons express receptors for DILPs and serotonin (Liu et al. 2015) and the Va neurons (CAPA1 and CAPA2) express CRZ receptors (Zandawala et al. 2019). **d** Cells expressing bursicon (Burs) activate cuticle plasticization and cuticle tanning (indirectly via DILP7-expressing neurons, DILP7n). Bursicon regulates its own release (Peabody et al. 2008). **e** Efferent peptidergic neurons innervate the hindgut and/or reproductive tract. Of the neurons shown, only those expressing DILP7 and PDF (thick arrows) have been analyzed functionally (Cognigni et al. 2011; Talsma et al. 2012; Yang et al. 2008). Thus, the functions of the remaining neurons are unknown (but see text)

Insects and other arthropods have segmental ganglia in the thorax and abdomen. In *Drosophila*, these ganglia are fused into one ganglionic mass, the thoracico-abdominal ganglia (or ventral nerve cord, VNC), resulting in three thoracic and nine abdominal neuromeres. In most insects studied, there are bilateral neurosecretory cells in each neuromere or ganglion (Nässel 1996; Nässel et al. 1994; Raabe 1989). The localization of peptidergic neurosecretory cells in the *Drosophila* VNC is shown in Fig. 9(a, b) and the cell types and peptides are listed in Table 2.

In flies, there are multiple types of neurosecretory cells in each abdominal neuromere (Fig. 9a, b), whereas in each of the thoracic neuromeres only one pair of cells has been identified (Fig. 9b). These thoracic cells, designated Tv neurons, express FMRFamide immunoreactivity and the gene encoding extended FMRFamides (Lundquist and Nässel 1990; Schneider et al. 1993a; Schneider et al. 1993b). The Tv neurons in *Drosophila* and other flies supply axon terminations to a plexus in the dorsal neural sheath over the entire ventral nerve cord (Lundquist and Nässel 1990; Nässel et al. 1988). In larvae, the six Tv neurons have axon terminations in segmental spherical neurohemal organs associated with dorsal median nerves (Nässel et al. 1988). In hemimetabolous insects, similar structures were named perivisceral organs or perisympathetic organs (see Predel 2001; Raabe 1989). So far, no other peptides have been identified in the Tv neurons or the dorsal axonal plexus (Nässel and Zandawala 2019; Predel et al. 2004; Wegener et al. 2006). Note that the mesothoracic neuromere has a second pair of FMRFamide-expressing ventral neurons (Fig. 9b) but it has not been established whether these supply axons to the dorsal neurohemal release site. The functional role of hormonal FMRFamide has not been clearly established in *Drosophila*, although it is known that the peptide modulates contractions in larval body wall muscles (Dunn and Mercier 2005; Hewes et al. 1998) and in the adult flight activity (Ravi et al. 2018). Wild-type flies respond to heat stress and certain infections by increased sleep; FMRFamide (and receptor) mutant flies display impaired sleep in response to these stressors (Lenz et al. 2015). In blowflies, FMRFamide stimulates secretion in salivary glands (Duve et al. 1992).

In abdominal neuromeres, there are several types of peptidergic neurosecretory cells in each neuromere (Fig. 9a, b). In neuromeres A2–A4, three pairs of Va neurons express Capa-gene products (CAPA1 and CAPA2 and CAPA-PK) (Kean et al. 2002; Terhzaz et al. 2015), 11 pairs of ABLKs produce LK (Cantera and Nässel 1992; de Haro et al. 2010) of which 3–4 anterior pairs co-express DH44 (Zandawala et al. 2018a), 4–5 pairs of cells produce GPA2/GPB5 (Sellami et al. 2011), six pairs express DH31 (Mandel et al. 2018), four pairs orcokinin A (OK-A) (Chen et al. 2015) and seven pairs produce bursicon (α and β) (Luan et al. 2006; Peabody et al. 2008). All of these neurosecretory cells have axons with terminations on surface of abdominal nerves and/or body wall muscle.

The functions of abdominal neurosecretory cells are shown in Fig. 9 (c–e). Several cells release peptides that regulate water and ion homeostasis (Fig. 9c). These peptides are LK, DH44, CAPA1 and CAPA2 and DH31 (Kean et al. 2002; Radford et al. 2002; Terhzaz et al. 1999; Terhzaz et al. 2015; Zandawala et al. 2018a). Orcokinin, GPB5, RYamide, and PDF might also play roles in diuresis (Sellami et al. 2011; Talsma et al. 2012; Veenstra and Khammassi 2017). Receptors for DH44, CAPA1 and CAPA2 and DH31 are expressed in principal cells, whereas the LK receptor is expressed in stellate cells of the Malpighian tubules, suggesting that these peptides regulate ion transport (see Nässel and Zandawala 2019). CAPA peptides from Va neurons act on the tubules to influence ionic and osmotic homeostasis thereby mediating desiccation tolerance and chill coma recovery (MacMillan et al. 2018; Terhzaz et al. 2015). CAPA neurons, in turn, are modulated by systemic CRZ from the LNCs. Abdominal neurons expressing LK, some of which also express DH44, are activated following water intake and likely stimulate diuresis and excretion (Zandawala et al. 2018a; Zandawala et al. 2018b). The hormonal functions of DH31 from abdominal neurosecretory cells are still not known (Mandel et al. 2018). Additionally, LK and GPB5 receptors are also expressed in the hindgut (Selcho et al. 2018; Zandawala et al. 2018a); however, the effects of LK and GPB5 on excretion or reabsorption by the hindgut have not yet been examined.

In pharate adult flies, bursicon and CCAP, together with DILP7-producing neurons, are involved in cuticle tanning and cuticle plasticization (wing expansion) (Fig. 9d). Bursicon, released from 14 abdominal neurons, stimulates wing expansion and cuticle tanning following adult ecdysis (Luan et al. 2006). The regulation of wing expansion by bursicon is hormonal, whereas the effect on cuticle tanning is mediated indirectly through DILP7-expressing neurons (transmitter unknown in tanning) (Flaven-Pouchon et al. 2020). Abdominal neurons expressing bursicon, in turn, are regulated by other inputs from the brain (likely CCAP), as well as potential autocrine feedback from bursicon that induces cell apoptosis (Peabody et al. 2008).

Several peptides are produced in efferent abdominal neurons that “innervate” the hindgut, or reproductive tract (Fig. 9e); neurons with AstA (Yoon and Stay 1995), proctolin (Anderson et al. 1988), DILP7 (Cognigni et al. 2011; Miguel-Aliaga et al. 2008), ITP (Dircksen et al. 2008), PDF (Nässel et al. 1993) and RYamide (Veenstra and Khammassi 2017) supply axons to the hindgut and rectum. DILP7 also supplies the female reproductive tract (Yang et al. 2008). The functional roles of these neurons have barely been investigated. PDF from abdominal efferents was shown to act at a distance on PDF receptor expressed on ureter muscles (at the base of Malpighian tubules) to induce contractions (Talsma et al. 2012), probably aiding secretion in the tubules. DILP7 acts in selection of egg-laying sites (Yang et al. 2008) and may also interact with IPCs to regulate food intake (Cognigni et al. 2011). For the other peptides, one might guess that they play roles in gut function, such as modulation of contractions (AstA and proctolin) and possibly water reabsorption (ITP and RYa).

## Intestinal peptides

The digestive tract of animals contains enteroendocrine cells (EECs), which represent another source of circulating hormones. Mammalian gut EECs express glucagon-like peptide-1 (GLP-1), gastric inhibitory polypeptide (GIP), ghrelin and cholecystokinin (CCK), which regulate various processes including regulation of hormone secretion, gut motility, nutrient homeostasis and feeding (Campbell and Drucker 2013; Gribble and Reimann 2019; Liddle 1997; Tong et al. 2010). Various dietary nutrients, as well as other hormones, trigger the EECs to release their contents into circulation. In *Drosophila*, the larval and adult midgut is a source of 11 different peptides (Table 2, Supplemental Fig. 2a) (Chen et al. 2016a; Reiher et al. 2011; Veenstra 2009; Veenstra et al. 2008; Veenstra and Ida 2014; Lemaitre and Miguel-Aliaga 2013). Some of these peptides are colocalized in different subpopulations of the EECs (Veenstra 2009; Veenstra et al. 2008; Veenstra and Ida 2014). In spite of the gut representing a rich source of peptides, studies investigating their functions in *Drosophila* are limited (Supplemental Fig. 2b). In adults, TK- and DH31-expressing EECs can be activated by dietary proteins and amino acids (Park et al. 2016; Song et al. 2014). One might predict that these peptides, as well as NPF, can influence ion and water transport through their receptors that are expressed in Malpighian tubules (Chintapalli et al. 2012; Coast et al. 2001; Söderberg et al. 2011). Furthermore, DH31 stimulates midgut muscle contractions (LaJeunesse et al. 2010), TK influences lipid production by the gut enterocytes (Song et al. 2014), bursicon alpha indirectly downregulates AKH signaling via Lgr2-expressing brain neurons (Scopelliti et al. 2014) and AstA, possibly from the gut EECs, reduces feeding and increases sleep (Chen et al. 2016b) (Supplemental Fig. 2b). In addition, larval CCHa2 targets IPCs and regulates food intake (Ren et al. 2015; Sano et al. 2015). Thus, functional studies on EEC-derived peptides are limited, largely due to the lack of tools to specifically and selectively target the signaling from the gut.

## Neurosecretory systems in the larval CNS

**Fig. 10:**
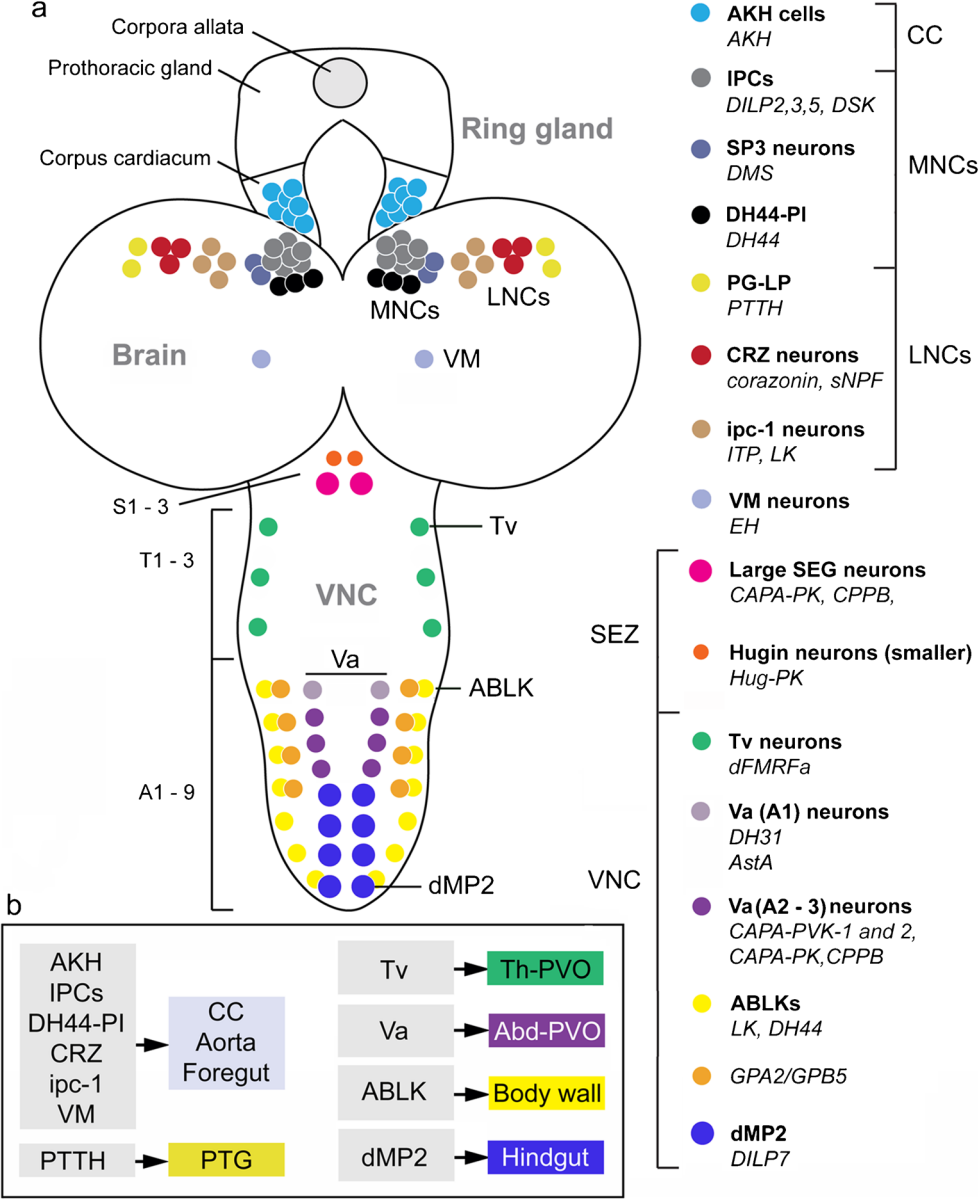
Neurosecretory cells in CNS of larval Drosophila. (a) Schematic depiction of cell bodies of neurosecretory cells in different regions of the CNS: corpora cardiaca (CC), median neurosecretory cells (MNC), lateral neurosecretory cells (LNC), subesophageal zone (SEZ; neuromeres S1–3) and ventral nerve cord (VNC; neuromeres T1–3 and A1–9). To the right, we display a legend of the different cell types (color coded) and their peptides (in bold the cell names, in italics the peptides). Note that the ipc-1 neurons are the same as the ITPn in the adult brain. The acronyms of the peptides are given in Table 1. (b) The primary release sites of different peptidergic hormone systems. Left column shows brain-derived hormones and right column hormones from cells in the VNC. Abbreviations: CC, corpora cardiaca; PTG, prothoracic gland; Th-PVO, thoracic perivisceral organs; Abd-PVO, abdominal perivisceral organs. This figure is updated and partly redrawn from a figure in Nässel and Zandawala et al. (2019), which in turn was based on Wegener et al. (2006)

In the *Drosophila* larva, the complement of peptidergic neurosecretory cells is rich (Fig. 10a). At this stage, the CC and CA, together with the prothoracic gland form a circular tissue, designated the ring gland, which surrounds the aorta (see Hartenstein 2006; Siegmund and Korge 2001). Clusters of LNCs and MNCs produce the same peptides as seen in adults, although the numbers of cells within the different clusters in some cases vary between the larva and adults. In addition, larvae have cells producing prothoracicotropic hormone (PTTH) and eclosion hormone (EH) (Fig. 10a), which undergo apoptosis after adult eclosion and are therefore no longer present in adult flies. The names of the neurosecretory cells with their peptides are given in Fig. 10(a). Larval neurosecretory cells of the brain have axon terminations in neurohemal areas in the ring gland, along the aorta and foregut (Siegmund and Korge 2001) (Fig. 10b). Most of the thoracic and abdominal neurosecretory cells send axons to neurohemal organs in dorsal median nerves (Fig. 10b), designated perivisceral organs (PVO), or perisympathetic organ (Predel 2001; Predel et al. 2003; Raabe 1989; Santos et al. 2007). Some of the cells (producing GPA1/GPB5, LK, and DH44) have axon terminations in body wall muscle or along the gut (dMP2 producing DILP7) (Fig. 10b) as shown in Cantera and Nässel et al. (1992), Miguel-Aliaga et al. (2008), Sellami et al. (2011) and Zandawala et al. (2018a). Not shown in Fig. 10 are efferent abdominal neurons innervating the hindgut that produce PDF, AstA and ITP (Dircksen et al. 2008; Nässel et al. 1993; Yoon and Stay 1995). Overviews of distribution of peptidergic neurons in the larval thoracico-abdominal ganglia are presented in Nässel and Zandawala et al. (2019), Park et al. (2008) and Santos et al. (2007).

As seen below, most of the larval neurosecretory cells play roles in developmental processes such as growth regulation, timing of developmental transitions and coordination of ecdysis motor behavior at molts. However, it is also likely that many of the systems regulate daily behavior and physiology. Such functions are barely studied in larvae.

**Fig. 11:**
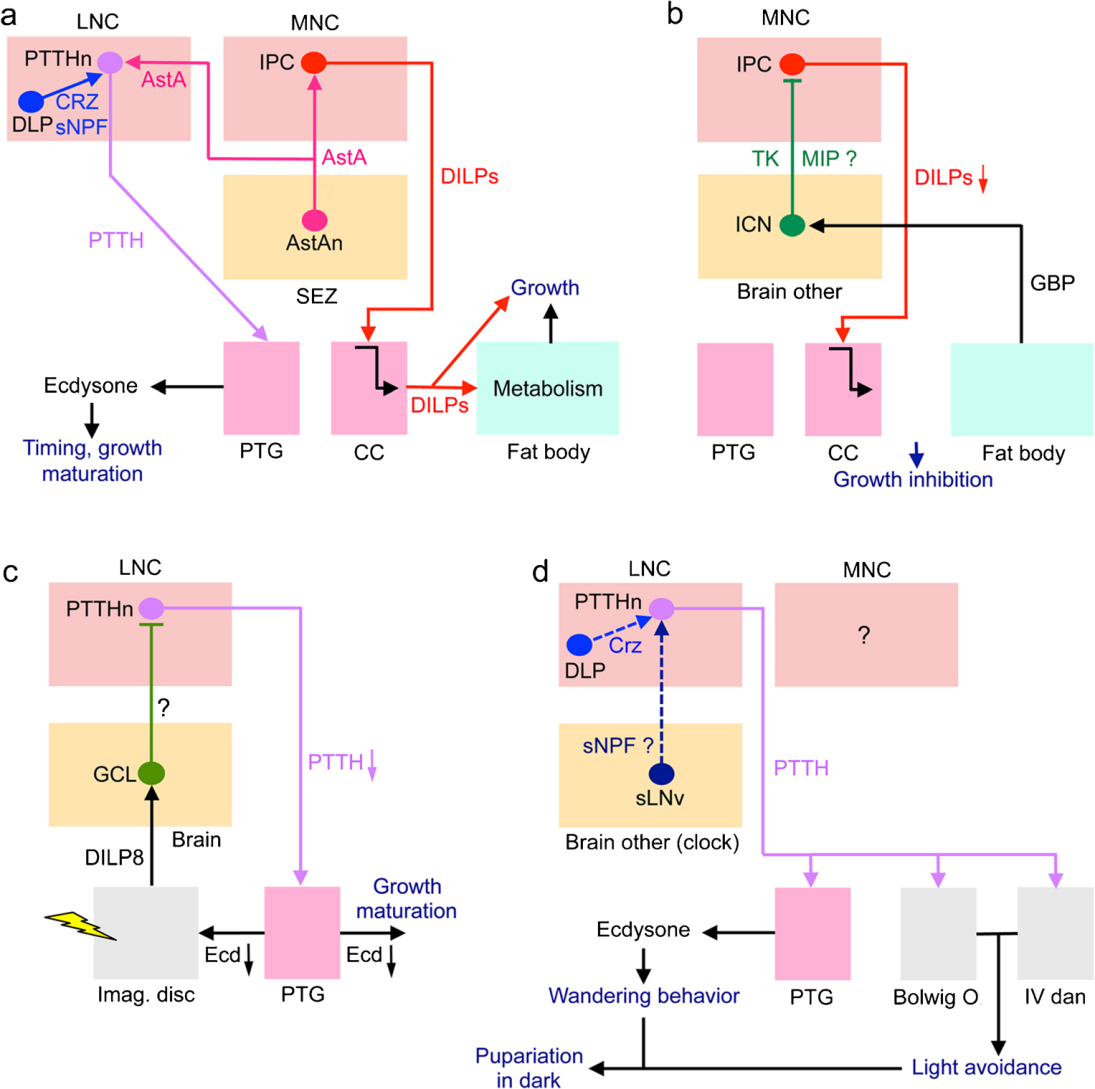
Schemes depicting neurosecretory systems in the larva. **a** Role of prothoracicotropic hormone neurons (PTTHn) and IPCs in timing of growth and maturation of third instar (L3) larvae. Neurons producing allatostatin A (AstAn) stimulate both PTTHn and IPCs via the receptor AstAR1 (DAR1) and this leads to ecdysone (Ecd) production and DILP release, which affects timing of development and maturation. In the mid L3, Ecd blocks growth and in late L3 the Ecd peak stimulates onset of sexual maturation (Deveci et al. 2019). Another study showed that corazonin (CRZ) activates PTTHn and thus basal Ecd production and increased larval growth (Imura and Shimada-Niwa 2020) and finally it was shown that both CRZ and sNPF from DLPs are required for regulation of IPCs (and growth) under nutrient restriction (Megha et al. 2019). It is not clear whether the two peptides (CRZ and AstA) cooperate in growth regulation since they were not investigated in the same study. **b** The IPCs are regulated by a pair of neurons (ICN) that produce tachykinin (TK) and myoinhibitory peptide (MIP) (Meschi et al. 2019). It was shown that TK inhibits IPCs and thus growth is inhibited. The ICNs are activated by growth-blocking peptide (GBP) from the fat body in a nutrient-dependent fashion. The role of MIP was not investigated. **c** Insulin-like peptide 8 (DILP8) is released upon damage to imaginal discs and acts on a set of four neurons (GCL) that express the DILP8 receptor Lgr3 (Colombani et al. 2015; Garelli et al. 2015; Vallejo et al. 2015). These GCL neurons inhibit production of PTTH by PTTHn and thus decrease growth and maturation of the larva. The transmitter of GCLs is unknown. **d** The late L3 larvae undergo a transition from feeding to wandering stages. At this point, they also become negatively phototactic. PTTH from the PTTHn acts on the prothoracic glands (PTG), the light-sensitive Bolwig organ and peripheral sensory neurons (class IV dendritic arborization neurons; IV dan) to alter light responses and via Ecd induce wandering behavior and finally pupariation in the dark (Yamanaka et al. 2013). The inputs to the sLNvs are from rhodopsin 6–expressing photoreceptors (not shown). In other studies, it was shown that the PTTHn are regulated with sNPF by the clock neurons sLNv (Selcho et al. 2017) and by CRZ-producing DLP neurons (Imura and Shimada-Niwa 2020). However, the direct link to the light avoidance/wandering behavior and pupariation is unclear (thus dashed lines). Another light-mediated pathway (not shown here but see a similar circuit in Supplementary Fig. 3) is provided by the PTTH neurons that signal to brain neurons producing eclosion hormone (EH) whose axons descend to the VNC where they contact motor neurons (Gong et al. 2019). A recent study also suggested that the NPF receptor is expressed in the PTG and that NPF signaling negatively regulates insulin signaling in the PTG influences and thereby affects growth and developmental timing (Kannangara et al. 2019). NPF was proposed to be acting systemically after release by EECs of the gut (not shown in Fig. 11)

Larval growth and maturation are mainly regulated by DILPs and PTTH (Fig. 11a–c). DILPs from the IPCs influence metabolism and consequently growth, through hormonal actions on the fat body and the nutrient-dependent TOR pathway (Fig. 11a) (see Brogiolo et al. 2001; Ikeya et al. 2002; Oldham and Hafen 2003). Both CRZ and sNPF from DLPs are required for regulation of IPCs (and growth) under nutrient restriction (Megha et al. 2019). PTTH, on the other hand, acts on the prothoracic glands via its receptor Torso to stimulate ecdysone production, which affects developmental timing and growth (Rewitz et al. 2009; Rewitz et al. 2013). Both the IPCs and PTTH neurons are stimulated by AstA (from the subesophageal zone) through its receptor DAR1 (Deveci et al. 2019). In addition, PTTH neurons are activated by CRZ from the DLPs and sNPF from the clock neurons sLNv (Fig. 11a) (Imura and Shimada-Niwa 2020; Selcho et al. 2017) and the IPCs are inhibited by TK from the ICN neurons, which are in turn activated by growth-blocking peptide (GBP) from the fat body in a nutrient-dependent fashion (Fig. 11b) (Meschi et al. 2019). The sLNv input to PTTH neurons is part of a circuit that synchronizes the central clock to that in the prothoracic gland and thereby times the eclosion (Selcho et al. 2017). A recent study also suggested action of NPF directly on the prothoracic gland to negatively regulate IIS and thus delay development (Kannangara et al. 2019). The authors proposed that NPF acts systemically after release by EECs of the gut. Another peptidergic pathway that might be involved in eclosion motor behavior is a set of PDF-expressing neurons (PDFtri) in the tritocerebrum that contact CCAP- and EH-producing neurons (Selcho et al. 2018) (Supplementary Fig. 3). These PDFtri neurons, which seem to undergo apoptosis after eclosion, may thus regulate/modulate EH release and thereby activate Inka cells to release ETH and induce ecdysis motor behavior (Selcho et al. 2018) but experimental evidence is yet to be provided. Finally, an additional level of growth coordination occurs through damage-induced DILP8 signaling from the imaginal discs, which via the intermediary Lgr3-expressing (DILP8 receptor) GCL brain neurons inhibits PTTH release and thus Ecd production and thereby delays growth and maturation (Fig. 11c) (Colombani et al. 2015; Garelli et al. 2015; Vallejo et al. 2015). This pathway is to ensure symmetric growth of the organism.

In addition to regulating growth and maturation, PTTH neurons are also part of the larval light-avoidance circuit (Fig. 11d) (Keene et al. 2011; Yamanaka et al. 2013). When the larvae reach the late 3rd instar stage, they cease feeding, stop being photophobic and begin wandering. Rhodopsin 6 (Rh6)–expressing photoreceptors of the Bolwig organ, when activated by light, relay signals to the clock neurons (LNvs) (Keene et al. 2011). The LNvs, in turn, activate PTTH neurons, which mediate light avoidance through hormonal actions of PTTH on the light-sensitive Bolwig organ and peripheral sensory neurons (class IV dendritic arborization neurons; IV dan) (Keene et al. 2011; Yamanaka et al. 2013). This behavioral switch is also mediated by PTTH-stimulated Ecd production by the prothoracic glands, thereby together with the altered light response enables the larvae to pupariate in the dark. It is not yet clear if CRZ-producing DLPs that lie upstream of PTTH neurons have any role in the light-avoidance behavior (Imura and Shimada-Niwa 2020). Another light-mediated pathway is mediated by the PTTH neurons that signal to brain neurons producing eclosion hormone (EH) whose axons descend to the VNC where they contact motor neurons (Gong et al. 2019). This pathway mediates a light-avoidance response during locomotion in the larva.

In summary, most studied functions of peptide hormones in larvae are related to development and growth but certainly some are likely to regulate locomotor behavior, feeding and metabolism, as well as diuresis and excretion (see Gong et al. 2019; Hückesfeld et al. 2016; Melcher and Pankratz 2005; Okusawa et al. 2014; Schoofs et al. 2014; Vanderveken and O'Donnell 2014; Yamanaka et al. 2013).

## Neurosecretory cells in other insects and crustaceans

There are not many comprehensive studies of peptide complement in specific brain neurosecretory cells of insects other than *Drosophila*. However, in *Manduca sexta*, *Aedes aegypti*, *Locusta migratoria* and especially *Rhodnius prolixus*, peptide immunocytochemistry has identified a number of such cells (Table 3, and references therein). In addition, mass spectrometry of dissected CC-CA complexes has confirmed many of these and identified additional peptides, also in the moth *Bombyx mori* (Supplementary material Table 1). It should be noted that most peptide data for *M. sexta* are old and derived from use of partly heterologous antisera (Zitnan et al. 1993) and that an update would be welcome. Somewhat surprisingly, only a few peptides were found in LNCs and MNCs of the studied insects (Table 3). In all insects studied to date, LNCs produce CRZ and PTTH (PTTH missing in adults in some insects) and MNCs express ILPs, myosuppressin and CRF-like DH (DH44 in *Drosophila*). Other peptides vary extensively between species. This common expression of several peptide hormones suggests that some ancient hormone functions have been evolutionarily conserved related to the control of feeding, metabolism, growth, reproduction and water homeostasis.

**Table 3:** Peptides in neurosecretory cells of other insects

Species^1^	LNC peptide	MNC peptide	Stage	Reference
*M. sexta*	ILP^2^	ILP^3^	Larva	(Mizoguchi et al. 1987; Zitnan et al. 1995)
	AstC, EH	Proctolin	Larva	(Zitnan et al. 1995)
	AstC, proctolin	DH (CRF)^4e^	Larva	(Zitnan et al. 1995)
	FMRFa^5^, PTTH	FMRFa^6^, RYa^7^	Larva	(Roller et al. 2016; Zitnan et al. 1995)
	ITP		Larva/adult	(Drexler et al. 2007)
	FMRFa^6^	FMRFa^6^, PDF	Adult	(Homberg et al. 1991)
	FMRFa^6^, proctolin	DH, PDF	Adult	(Homberg et al. 1991)
*Ae. aegypti* ^*8*^	CCAP	ILPs	Adult	(Strand et al. 2016)
	Corazonin	MS	Adult	(Strand et al. 2016)
		NPF	Adult	(Strand et al. 2016)
		OEH^9^	Adult	(Strand et al. 2016)
*R. prolixus*	AstA		Larva (5th)	(Sarkar et al. 2003; Zandawala et al. 2012)
	CAPA		Larva (5th)	(Paluzzi and Orchard 2010; Paluzzi et al. 2008)
	Corazonin		Larva/adult	(Patel et al. 2014)
	DH31	DH31	Larva (5th)	(Te Brugge et al. 2005; Zandawala et al. 2011)
	Orcokinin A		Larva (5th)	(Wulff et al. 2017)
	PTTH		Larva/adult	(Vafopoulou et al. 2007)
		ILPs	Larva (5th)	(Defferrari et al. 2016)
		Allatotropin	Larva (5th)	(Masood and Orchard 2014)
		DH (CRF)	Larva (5th)	(Te Brugge et al. 2001)
		FMRFa^6^	Larva (5th)	(Tsang and Orchard 1991)
		LK	Larva (5th)	(Te Brugge et al. 2001)
		MIP	Adult	(Lange et al. 2012)
		NPF	Larva (5th)	(Gonzalez and Orchard 2008; Sedra and Lange 2016)
		SIFa^10^	Larva/adult	(Ayub et al. 2020)
*L. migratoria*		ILP	Adult	(Goltzené et al. 1992; Lagueux et al. 1990)
		Neuroparsins	Adult	(Goltzené et al. 1992; Tamarelle and Girardie 1989)
		DH (CRF)	Adult	(Patel et al. 1994)
		ACP^11^	Adult	(Clynen and Schoofs 2009)
		AstA^11^	Adult	(Clynen and Schoofs 2009)
		MS^11^	Adult	(Clynen and Schoofs 2009)
		sNPF^11^	Adult	(Clynen and Schoofs 2009)
*Consensus*^12^	CRZ	ILPs		
	PTTH	MS		
		DH (CRF)		

Peptide acronyms as in Table 1

^1^The species are *Manduca sexta*, *Aedes aegypti*, *Rhodnius prolixus* and *Locusta migratoria*

^2^ILP, insulin-like peptide (bombyxin); the cells colocalize proctolin and FMRFa

^3^The cells colocalize proctolin

^4^DH (CRF), corticotropin-releasing factor like diuretic hormone

^5^Antiserum to FMRFamide, probably myosuppressin in these cells (Yamada et al. 2017)

^6^FMRFa could mean products from any of 5–6 peptide precursor genes

^7^RYamide possibly colocalized in one pair (Roller et al. 2016)

^8^The cited review does not specify species in their figure, so could also include *Anopheles gambiae*. Note that the peptides listed were also found in the corpora cardiaca, suggesting they are all hormones (or some are release regulators)

^9^OEH, ovary ecdysteroidogenic hormone

^10^Appears to be regular SIFa interneurons (in PI) that supply axon terminations to retrocerebral complex

^11^These peptides were determined in dissected pars intercerebralis by mass spectrometry

^12^Only a few peptides were found in common in LNCs and MNCs in these species and *Drosophila*

In the VNC, there are also some conserved peptidergic neurosecretory cell systems. For instance, pairs of LK (Cantera et al. 1992; Chen et al. 1994; Nässel et al. 1992; Te Brugge et al. 2001)- and CAPA (Kean et al. 2002; Loi and Tublitz 2004; Paluzzi et al. 2008; Predel and Wegener 2006)-producing neurosecretory cells have been shown laterally in abdominal neuromeres in a number of species from different insect orders. Furthermore, sets of pyrokinin/PBAN (pheromone biosynthesis activating neuropeptide) expressing cells have been identified in the SEZ of several insect species (Choi et al. 2001; Davis et al. 1996; Hellmich et al. 2014; Meng et al. 2002; Sato et al. 1994).

In *Drosophila melanogaster*, the peptidome is somewhat reduced compared to species in basal insect orders but also compared to some other *Drosophila* species (Liessem et al. 2018; Nässel and Zandawala 2019; Veenstra 2010; Veenstra and Khammassi 2017). For instance, vasopressin-like peptide (inotocin), AKH/corazonin-related peptide (ACP), allatotropin, calcitonin, elevenin, neuroparsin, parathyroid hormone–like peptide and TRH genes have been lost (Nässel and Zandawala 2019; Veenstra and Šimo 2020; Xie et al. 2020). This may suggest that in *Drosophila*, other peptides have taken over the roles of the missing ones (see examples of JH regulation below). Systematic comparative studies of neuropeptide and peptide hormone functions in insects with larger and reduced peptidomes are required to resolve this.

One example of interesting differences between insects of different taxonomic groups is the regulation of JH production. Although JH displays vital functions during development and in adult reproduction and physiology in insects (Riddiford 2008; Truman et al. 2006; Truman and Riddiford 2019), its production is regulated by different unrelated neuropeptides encoded by distinct genes. Hence, in cockroaches, crickets and termites, AstA is a primary inhibitor of JH production (Bendena et al. 2020; Woodhead et al. 1989); in the cricket *Gryllus bimaculatus*, AstB (MIP) is a regulator (Lorenz et al. 1995); and in moths, mosquitos and flies, e.g., *Drosophila*, AstC is a JH inhibitor (Bendena et al. 2020; Kramer et al. 1991; Wang et al. 2012b). Thus, although all three peptide types are present in *Drosophila*, only AstC seems to affect JH production and it was suggested that the peptide is derived from MNCs innervating the CA (Kreienkamp et al. 2002; Zitnan et al. 1993) but clear data were missing. The stimulators of JH production also vary. In several species, including moths where it was first discovered (Kataoka et al. 1989a), an allatotropin (AT) has been identified but it is missing in *Drosophila* (see Bendena et al. 2020). In the moth *Bombyx*, AT acts indirectly via sNPF neurons in CA (Yamanaka et al. 2008). In some mosquitos, AT may act directly on JH production (Li et al. 2003) but in *Aedes aegypti* not only AT but also another peptide, ecdysis-triggering hormone (ETH), from epitracheal (Inka) cells increases JH biosynthesis (Areiza et al. 2014). In *Drosophila* where AT is missing, ETH from Inka cells was also found to stimulate JH production and thereby diminish ovary maturation (Meiselman et al. 2017). Additionally, there are reports suggesting that DILPs, via the dInR, regulate JH production in CA of adult *Drosophila* (Belgacem and Martin 2007; Rauschenbach et al. 2014; Tatar et al. 2001; Tu et al. 2005). In conclusion, it appears that hormonal regulation is to some extent plastic and that specific functions can be performed in different organisms by utilizing different messengers. The peptides discussed above are furthermore functionally highly pleiotropic and probably several of their functions are taxon specific (see Bendena et al. 2020; Nässel and Zandawala 2019).

It could be noted here that in another large group of arthropods, the crustaceans, the studies of neurosecretory cell systems have focused on the X-organ-sinus gland (XO-SG) in the eyestalks but also to some extent the anterior cardiac plexus (and anterior commissural organ) of the stomatogastric nervous system and the pericardial organs (POs) associated with the thoracic ganglia (see Christie 2011). Whereas the XO-SG could be reminiscent of the LNC-CC in insects, no analogs of the MNCs have been proposed. Quite a few peptides have been identified in the SG of decapod crustaceans, including sNPF, TK and the ITP-like crustacean hyperglycemic hormone (CHH) (Christie 2011), which are also found in *Drosophila* LNCs. Other SG peptides are myosuppressin, orcokinin, orcomyotropin, proctolin, the AKH-like red pigment concentrating hormone and SIFamide (Christie 2011). Hence, the XO-SG shares peptides also with PI/MNCs and CC of insects. For a comprehensive list of peptides in decapod crustaceans, see Veenstra (2016).

## Do insect neurosecretory cell systems share functions with those in mammals?

**Fig. 12:**
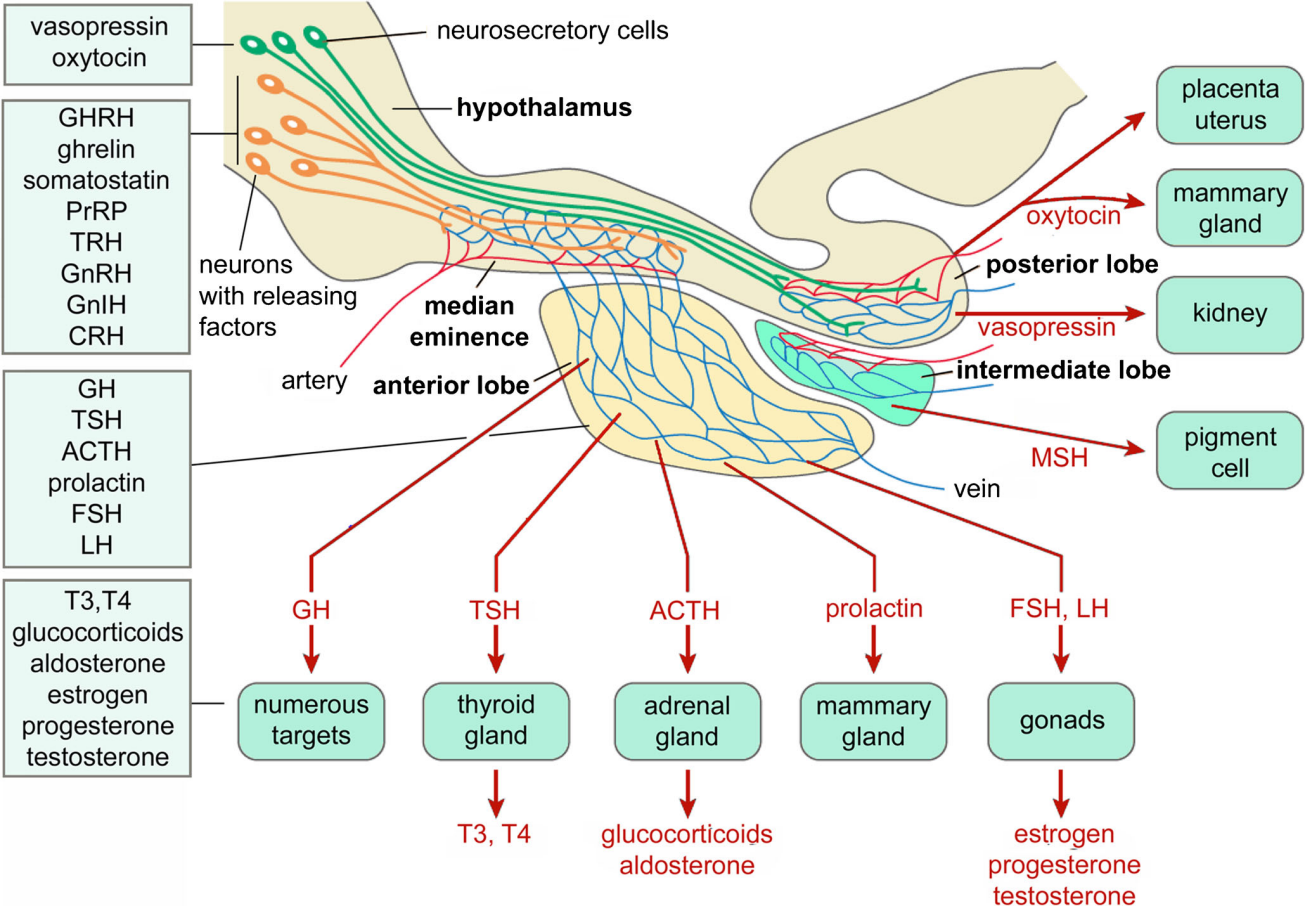
Signaling in hypothalamus and pituitary in mammals. This scheme presents the most common factors seen in the hypothalamus-pituitary axes of mammals. The intermediate pituitary lobe (with its separate capillary network) and MSH signaling are not found in humans and many other mammals. Peptides are released from hypothalamic neuroendocrine cells into separate capillary networks. Sets of neuroendocrine cells release neuropeptides to the anterior lobe of the pituitary via capillaries in the median eminence. These peptides regulate the release of pituitary peptide hormones produced by endocrine cells of the anterior lobe (these cells are not shown). Additionally, dopamine and GABA exert influence on the endocrine cells (not shown). The posterior capillary network receives peptide hormones (oxytocin and vasopressin) from large hypothalamic neurons whose axons project to the posterior lobe of the pituitary. These peptides are released into the general circulation that transports them to distant target organs. The different hormones produced in the anterior lobe are also released into circulation and act on multiple targets. Some target organs (thyroid, adrenal and gonads) in turn produce “secondary” hormones of different kinds. Some of these hormones provide feedback to the hypothalamus. Abbreviations: GHRH, growth hormone–releasing hormone; PrRP, prolactin-releasing peptide; TRH, thyrotropin-releasing hormone; GnRH, gonadotropin-releasing hormone; GnIH, gonadotropin-inhibiting hormone, CRH, corticotropin-releasing hormone; GH, growth hormone; TSH, thyroid-stimulating hormone; ACTH, adrenocorticotropic hormone; FSH, follicle-stimulating hormone; LH, luteinizing hormone; T3, triiodothyronine; T4, thyroxine; MSH, melanocyte-stimulating hormone. This figure is updated from Nässel and Larhammar (2013), with permission

**Fig. 13:**
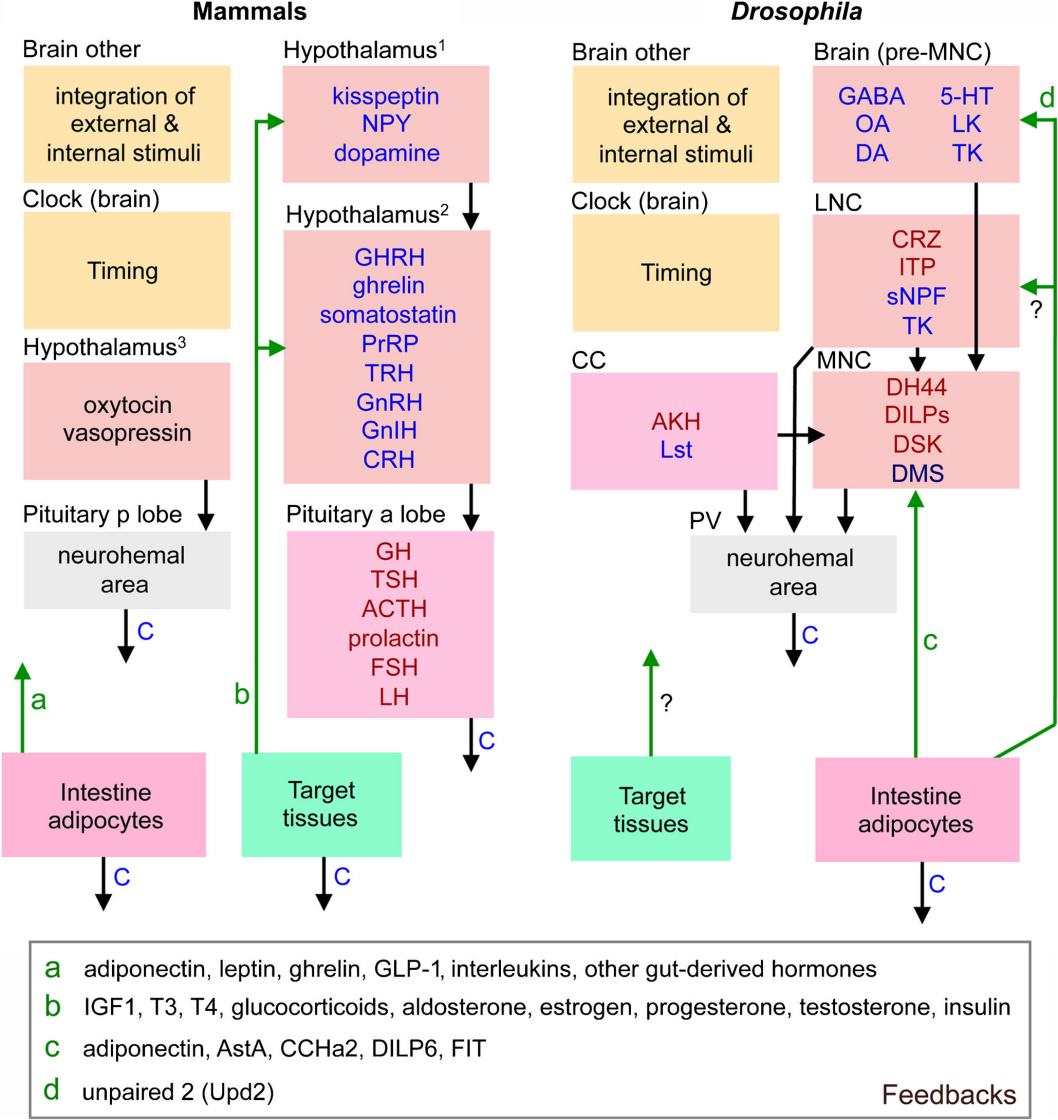
Comparison between mammalian neurosecretory systems and those of *Drosophila*. This highly schematic figure shows the key features of the brain-hypothalamus-pituitary axis in comparison with presumably analog regions in *Drosophila*. Same colors indicate presumed similar regions. Hypothalamus is divided into three regions (1–3) and the pituitary into anterior (a) and posterior (p) lobes. Substances indicated in blue are neuromodulators and/or release-regulating factors, those in dark red are hormones and the function of dromysosuppressin (DMS) in MNCs is not clear although the DMS-MNC cells have axons terminating together with those of the other MNCs. Arrows indicate action of the substances on cells in a region, blue C:s indicate release into general circulation and green arrows are feedback from target tissues. The regions “brain other” and “clock” are not interconnected to other regions with arrows to avoid complexity but certainly play important roles in regulating neurosecretory systems (and receive feedback from hormonal systems). We lumped together intestine and adipocytes as one set of target tissues and other targets are grouped in target tissues (these are, e.g., gonads, thyroid, adrenals and liver in mammals and gonads and muscles in *Drosophila*). Adipocytes in *Drosophila* include other fat body functions (e.g., liver-like and immune functions). Substances used as feedback are indicated by green (a–d) and are listed in the box below (GLP-1, glucagon-like peptide 1; IGF1, insulin-like growth factor 1; T3, triiodothyronine; T4, thyroxine; FIT, female-specific independent of transformer). Acronyms of other factors and hormones are as in Table 1. Other abbreviations: CC, corpora cardiaca; MNC, median neurosecretory cells; LNC, lateral neurosecretory cells; PV, proventriculus region (including aorta, crop duct, and CC)

As mentioned, it has been suggested for quite some time that the insect brain neuroendocrine system bears similarities to those in the vertebrate hypothalamus-pituitary-adrenal (HPA), hypothalamus-pituitary-thyroid (HPT) and hypothalamus-pituitary-gonadal (HPG) axes (Hartenstein 2006; Scharrer 1987; Scharrer and Scharrer 1963). It has also been shown that many of the peptides and peptide receptors of insects and mammals are ancestrally related (Jekely 2013; Mirabeau and Joly 2013). Yet anatomical and functional analogies between the hormonal systems of these taxa are not that straight forward. As seen in Figs. 2, 12 and 13, there are some basic similarities but also numerous differences. Only a few of the mammalian HPA peptides/peptide hormones are shared with those found in insects: vasopressin-inotocin, somatostatin-allatostatin C, GnRH-AKH/CRZ, TRH (thyroid-stimulating hormone–releasing hormone, or thyrotropin-releasing hormone)-EFLamide (TRH-like peptide) and CRF-DH44. Thus, many hypothalamus-pituitary (HP) peptides seem not to exist in insects and furthermore *Drosophila* has neither inotocin nor TRH-like peptide (see Liutkeviciute et al. 2016; Nässel and Zandawala 2019; Odekunle and Elphick 2020; Veenstra and Šimo 2020). It is also interesting to note that inotocin, the insect ortholog of the antidiuretic hormone vasopressin, is produced by interneurons in insects and seems not to function as a circulating hormone, although it can to act indirectly to trigger release of a diuretic factor that stimulates secretion in Malpighian tubules (Aikins et al. 2008; Stafflinger et al. 2008). Also TRH functions seem to have diverged over evolution since the distribution of EFLamide in locusts suggests an interneuronal function in regulation of the central complex circuits and that a hormone-releasing function as seen in vertebrates is not likely in insects (Veenstra and Šimo 2020). Hence, only CRF-DH44, GnRH-AKH and possibly somatostatin-allatostatin C remain as peptides shared by the HP and LNC/MNC systems.

Another difference is that in mammals the majority of the pituitary hormones (at least six) are produced by glandular cells in the anterior pituitary and released into circulation via the vasculatory system (Figs. 12, 13), whereas in insects, only two peptide hormones are produced by CC glandular cells in AKH and Lst (Fig. 13). In contrast, quite a few LNC- and MNC-derived peptide hormones are released (from neurosecretory cells) directly into circulation via neurohemal areas, whereas in mammals only vasopressin and oxytocin are released from hypothalamic neurosecretory cells (Figs. 12, 13). Furthermore, in mammals, a number of peripheral glands and tissues throughout the body that are targeted by pituitary hormones produce several “secondary” hormones (Fig. 12). In insects, only a few target tissues/glands (e.g., CA, prothoracic glands and gonads) are known to produce hormones such as Ecd and JH. However, the insect fat body, a liver and adipocyte analog, is targeted by several brain-derived hormones and produces a number of factors acting on the CNS neurosecretory system, similar to mammalian adipocytes (Fig. 13).

The general functions of a few insect LNC-MNC peptides partly overlap with those of some HP peptides: (1) DILPs produced by MNCs regulate growth (in larvae/pupae); (2) ion/water homeostasis is regulated by ITP and DH44; (3) metabolism by CRZ, ITP, DILPs and DH44 (and AKH from CC); and (4) gonad maturation and reproduction by DILPs (aspects of reproduction by DH44 and CRZ). In mammals, these functions are served by (1) growth hormone, (2) vasopressin, (3) TSH (and T3, T4) and (4) FSH and LH (see Figs. 12, 13). Possibly, CRZ and DILPs (from DLPs and IPCs) are part of an axis regulating stress responses like the HPA axis in mammals (see Figs. 12, 13). Since there are no adrenal glands in insects, the corresponding secondary hormones are not known in insects. Perhaps octopamine (OA) and dopamine (DA), as well as Ecd and JH, are part of the stress signaling downstream of DILPs and CRZ (Gruntenko et al. 2016; Gruntenko et al. 2005; Gruntenko et al. 2010; Lubawy et al. 2020; Petruccelli et al. 2020; Roeder 2020; Zhao et al. 2010). Thus, the cellular sources of insect stress factors are in adults spread out into CA (JH), sets of neurons (OA, DA) and gonads (Ecd). Other peptide hormones, including modulators of water/ion homeostasis and metabolism, have been implicated in responses to insect cold stress where hemolymph osmolarity is a critical factor for survival (Lubawy et al. 2020; Overgaard and MacMillan 2017; Terhzaz et al. 2015). The water/ion homeostasis is compromised during exposure to low temperature and the recovery (chill coma recovery) involves a resetting of osmotic and ionic balance. Thus, peptides such as CAPA (MacMillan et al. 2018; Terhzaz et al. 2015), ILPs (Broughton et al. 2005; Lingo et al. 2007; Nässel and Vanden Broeck 2016) and maybe DH31, DH44, ITP and LK (Cannell et al. 2016; Li et al. 2020; Lubawy et al. 2020) are likely to be of importance in regulation of cold-induced stress. Recently, a link between CRZ and CAPA in regulation of chill coma recovery was discovered, suggesting that DLPs and CAPA producing Va neurons in the VNC constitute another stress-signaling axis (Zandawala et al. 2020).

Thus, in summary, the anatomical and functional organization of postembryonic brain neurosecretory systems seems to have diversified over evolution. Yet there seems to be also some convergent evolution of hormone function. For example, mammalian glucagon released from the pancreas and the evolutionary unrelated AKH of CC seem to be functional analogs in carbohydrate mobilization (Kim and Rulifson 2004; Lee and Park 2004). On the other hand, mammalian insulin and insect ILPs are ancestrally related and share many functions but the cell systems releasing them are very different.

Another interesting difference between insects and mammals is the large number of neurosecretory cells (and peptide hormones) associated with the abdominal ganglia in insects (Fig. 10) and the lack of such systems associated with the spinal cord. In fish, however, there is a caudal neurosecretory system in the posterior spinal cord with so-called Dahlgren cells supplying axon terminations to an associated neurohemal area, the urophysis (see Fridberg and Bern 1968; Nässel and Larhammar 2013). The neurosecretory Dahlgren cells produce the antidiuretic peptides isotocin and arginine vasopressin, as well as other osmoregulatory hormones such as urotensin I, urotensin II, corticotropin-releasing factor (CRF) and parathyroid hormone–related protein (Gozdowska et al. 2013; Ingleton et al. 2002; Lederis et al. 1982; Pearson et al. 1980). Thus, the fish urophysis appears to share functions with the insect caudal neurosecretory cells that produce LK, DH44, DH31, ITP and GPA2/GPB5, known to regulate water and ion homeostasis.

## Conclusions and future perspectives

We have outlined neurosecretory and peptidergic systems in *Drosophila* and provided some comparisons with other insects as well as vertebrates. The regulation of *Drosophila* development, behavior and physiology relies on both (1) peptide hormones from LNCs, MNCs and other neurosecretory cells; (2) regulatory peptidergic interneurons that are part of high level neuronal circuits; and (3) peptidergic (and other) interneurons that regulate release of peptide hormones (sometimes in addition to being part of circuits). Hormonal axes in *Drosophila* are morphologically less distinctly organized (cells are more distributed) than those of the different hypothalamus-pituitary axes of vertebrates. Nevertheless, we can outline hormonal axes in *Drosophila* that regulate metabolism, appetite and food intake, reproductive behavior and physiology, sleep and activity rhythms and water and ion balance (excretion), as well as different developmental processes.

The *Drosophila* peptidome is somewhat reduced compared to several other insect species, suggesting that some peptide hormone functions are missing or taken over by other peptides. It would therefore be of great interest to widen the studies of insect peptide hormone functions and to explore other non-model insects with larger peptidomes. There are several *Drosophila* peptides whose adult functions are poorly known (for instance AstC, orcokinin, CCAP, CCHa2, proctolin and FMRFamide) and for some it even remains to be determined whether they have hormonal roles or not (for instance AstC, orcokinin, DH31 and CCHa2). With the multitude of novel imaging techniques available (see Bates et al. 2020), it would furthermore be important to explore the peptidergic and neurosecretory systems to determine interactions between different regulatory systems. In this context, it is for instance rarely known how sensory inputs influence neurosecretory systems, except for systems driven by nutrient sensing. Thus, although there are detailed studies of neurosecretory systems regulating water and ion homeostasis, we barely know how sensory information is relayed to the relevant neurosecretory cells. Also, feedback from target tissues to neurosecretory cells in the CNS are little explored, except for signals from the fat body (Owusu-Ansah and Perrimon 2015). Thus, insect endocrinology, although it has a long history, seems in many ways to be in its infancy.

## Electronic supplementary material

ESM 1 (PDF 1246 kb)

## Footnotes

1 See Neuropeptide database: http://www.neuropeptides.nl
