# Supplementary material for: Hormonal axes in Drosophila: regulation of hormone release and multiplicity of actions
Source: Cell Tissue Res. 2020 Aug 22;382(2):233–66. doi: 10.1007/s00441-020-03264-z (PMC7584566; doi:10.1007/s00441-020-03264-z)
Supplement: Supplementary file 1 — (PDF 1246 kb) [file 441_2020_3264_MOESM1_ESM.pdf]

Supplementary Material Files

Hormonal axes in *Drosophila:* regulation of hormone release and multiplicity of actions

Dick R.Nässel[1] and Meet Zandawala[2]

Supplementary Table 1. Neuropeptides detected by mass spectrometry of dissected glands in *Bombyx mori* and *Locusta migratoria*.

Species	CC/CA Peptide	Stage	Reference
Bombyx mori	AKH	Larva	(Liu et al., 2012)
	AstA	Larva	
	AstC	Larva	
	Allatotropin	Larva	
	BRFa[1]	Larva	
	BMS	Larva	
	CAPA-PVK	Larva	
	CCAP	Larva	
	CRZ	Larva	
	NPLP1	Larva	
	Orcokinin	Larva	
	PTST (MIP) [1]	Larva	
	SGNP (PRLa)	Larva	
	sNPF[1]	Larva	
	SK	Larva	
	TK[1]	Larva	
Locusta migratoria	PK1-7 [2]	Adult	(Clynen and Schoofs, 2009)
	Lom-AG-MT-2 [2]	Adult	
	FLRFa (MS) [2]	Adult	
	sNPF [2]	Adult	
	AstA4,5,9,10 [2]	Adult	
	NPF [3]	Adult	
	CRZ [3]	Adult	
	LomHrTH (ACP) [3]	Adult	
	AKH I-III [3]	Adult	
Carausius morosus	AKH	Adult	(Liessem et al., 2018)
	CRZ	Adult	
	MIP7,9,11	Adult	
	PK1,7,9	Adult	
	MS	Adult	
	NPLP-1	Adult	
	sNPF	Adult	
	PKL1,2[4]	Adult	
	RFLa[4]	Adult	
	HanSolin[4]	Adult	

Notes

Peptide acronyms as in Table 1

[1] These peptides might act as release regulating factors (BRFa, *Bombyx* RFamide; PTST, AstB and MIP; others as in Table 1)

[2] In both CC and CA

[3] Only in CC

[4] Novel peptides, first detected in *C. morosus*

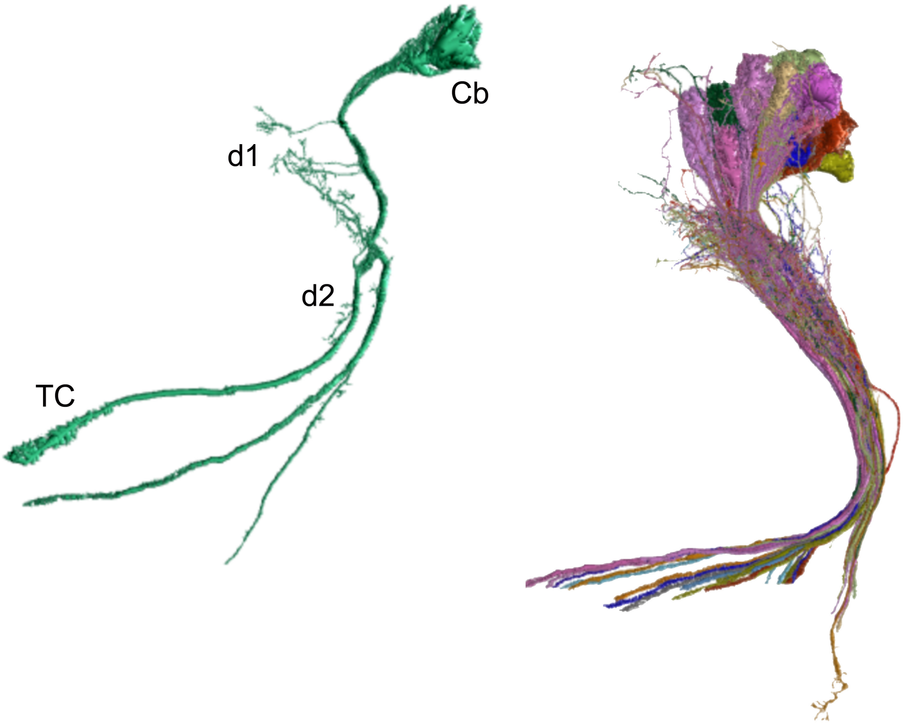

S. Fig. 1. *Brain insulin producing cells (IPCs) reconstructed from serial electron microscopic sections of a Drosophila hemibrain*. To the left side view of one IPC, to the right a cluster of IPCs in similar view. Cb, cell body; d1 and d2 two sets of presumed dendrites. This figure was compiled from data in neuPRINT (https://neuprint.janelia.org) (Clements et al., 2020; Xu et al., 2020; Zheng et al., 2018).

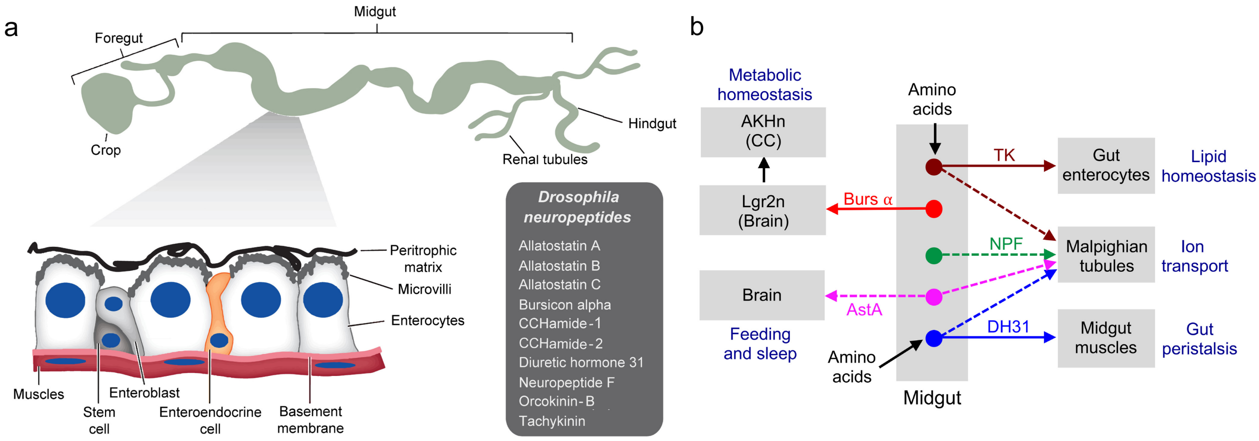

S. Fig. 2. *Neuropeptides in enteroendocrine cells (EECs) of the intestine.* **a.** A scheme depicting the organization of the digestive tract in adult *Drosophila*. The *Drosophila* gut spans across the entire thorax and abdomen and is comprised of the foregut, midgut, Malpighian (renal) tubules and hindgut. The lower part illustrates that the midgut is composed of five different cell types: muscle cells, stem cells, enteroblasts, enterocytes and enteroendocrine cells. Ten neuropeptides/peptide hormones expressed in the adult *Drosophila* enteroendocrine cells are listed in the box. This figure is from (Nässel and Zandawala, 2019), which was based on (Lemaitre and Miguel-Aliaga, 2013). **b.** Schematic depiction of signaling from EECs. Solid arrows indicate that experimental data is available for function, and dashed arrows depict hypothetical functions. See text for details, and references. AKHn, AKH producing cells in CC; Lgr2, bursicon receptor; other acronyms in Table 1.

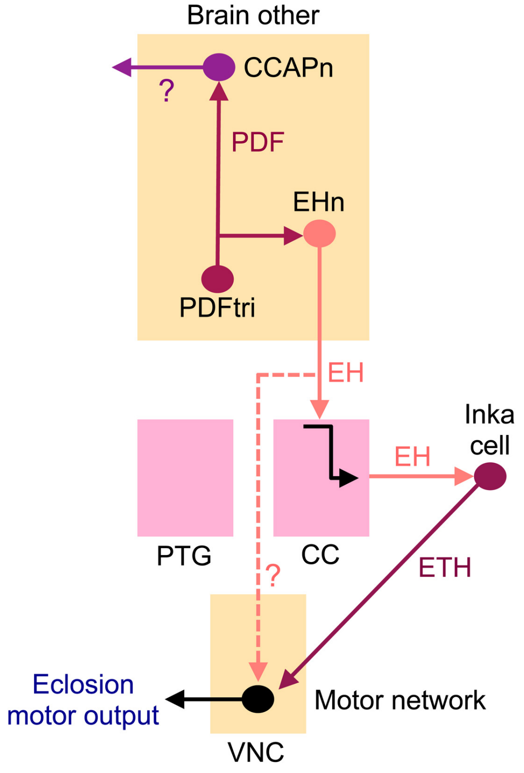

S. Fig. 3. *Pigment-dispersing hormone (PDF) in tritocerebral neurons (PDFtri).* A set of PDFtri neurons contact CCAP- (CCAPn) and EH producing (EHn) neurons and may be part of a circuit regulating adult eclosion via Inka cells and a motor network in the VNC (Selcho et al., 2018). This scheme is based on anatomy (GRASP technique) only and experimental data is required to verify the circuit.

References

Clements J, Dolafi T, Umayam L, Neubarth NL, Berg S, Scheffer LK, Plaza SM (2020) *neu*Print: Analysis Tools for EM Connectomics. bioRxiv:2020.2001.2016.909465

Clynen E, Schoofs L (2009) Peptidomic survey of the locust neuroendocrine system. Insect Biochem Molec 39:491-507

Lemaitre B, Miguel-Aliaga I (2013) The Digestive Tract of Drosophila melanogaster. Annual Review of Genetics, Vol 47 47:377-404

Liessem S, Ragionieri L, Neupert S, Büschges A, Predel R (2018) Transcriptomic and Neuropeptidomic Analysis of the Stick Insect, *Carausius morosus*. J Proteome Res 17:2192-2204

Liu X, Ning X, Zhang Y, Chen W, Zhao Z, Zhang Q (2012) Peptidomic Analysis of the Brain and Corpora Cardiaca-Corpora Allata Complex in the *Bombyx mori*. International journal of peptides 2012:640359

Nässel DR, Zandawala M (2019) Recent advances in neuropeptide signaling in Drosophila, from genes to physiology and behavior. Progr Neurobiol 179:101607

Selcho M, Muhlbauer B, Hensgen R, Shiga S, Wegener C, Yasuyama K (2018) Anatomical characterization of PDF-tri neurons and peptidergic neurons associated with eclosion behavior in Drosophila. J Comp Neurol 526:1307-1328

Xu CS, Januszewski M, Lu Z, Takemura S-y, Hayworth KJ, Huang G, Shinomiya K, Maitin-Shepard J, Ackerman D, Berg S, Blakely T, Bogovic J, Clements J, Dolafi

T, Hubbard P, Kainmueller D, Katz W, Kawase T, Khairy KA, Leavitt L, Li PH, Lindsey L, Neubarth N, Olbris DJ, Otsuna H, Troutman ET, Umayam L, Zhao T, Ito M, Goldammer J, Wolff T, Svirskas R, Schlegel P, Neace ER, Knecht CJ, Alvarado CX, Bailey DA, Ballinger S, Borycz JA, Canino BS, Cheatham N, Cook M, Dreher M, Duclos O, Eubanks B, Fairbanks K, Finley S, Forknall N, Francis A, Hopkins GP, Joyce EM, Kim S, Kirk NA, Kovalyak J, Lauchie SA, Lohff A, Maldonado C, Manley EA, McLin S, Mooney C, Ndama M, Ogundeyi O, Okeoma N, Ordish C, Padilla N, Patrick C, Paterson T, Phillips EE, Phillips EM, Rampally N, Ribeiro C, Robertson MK, Rymer JT, Ryan SM, Sammons M, Scott AK, Scott AL, Shinomiya A, Smith C, Smith K, Smith NL, Sobeski MA, Suleiman A, Swift J, Takemura S, Talebi I, Tarnogorska D, Tenshaw E, Tokhi T, Walsh JJ, Yang T, Horne JA, Li F, Parekh R, Rivlin PK, Jayaraman V, Ito K, Saalfeld S, George R, Meinertzhagen I, Rubin GM, Hess HF, Scheffer LK, Jain V, Plaza SM (2020) A Connectome of the Adult *Drosophila* Central Brain. bioRxiv:2020.2001.2021.911859

Zheng Z, Lauritzen JS, Perlman E, Robinson CG, Nichols M, Milkie D, Torrens O, Price J, Fisher CB, Sharifi N, Calle-Schuler SA, Kmecova L, Ali IJ, Karsh B, Trautman ET, Bogovic JA, Hanslovsky P, Jefferis GSXE, Kazhdan M, Khairy K, Saalfeld S, Fetter RD, Bock DD (2018) A Complete Electron Microscopy Volume of the Brain of Adult *Drosophila melanogaster*. Cell 174:730-743.e722
